# Supplementary material for: Community-based interventions targeting multiple forms of malnutrition among adolescents in low-income and middle-income countries: protocol for a scoping review
Source: BMJ Open. 2024 Mar 28;14(3):e078969. doi: 10.1136/bmjopen-2023-078969 (PMC10982731; doi:10.1136/bmjopen-2023-078969)
Supplement: Supplementary data [file bmjopen-2023-078969supp001.pdf]

Supplementary File 1. Search strategies in 3 different databases

**Community-based interventions targeting multiple forms of malnutrition among adolescents in low- and middle-income countries: protocol for a scoping review**			
**No.**	**Concept**	**PubMed search terms**	**Number of records (As per 14th July)**
#1	**Randomized controlled trial Controlled before-after studies Quasi experimental studies**	("randomized controlled trial"[pt] OR "random allocation"[mesh] OR "cross-over studies"[mesh] OR "Controlled Before-After Studies"[Mesh] OR quasi experiment*[tiab] OR quasiexperiment*[tiab] OR “quasi-experiment*”[tiab])	720,799<br>556,703 (2000-2023)
#2	**Nutrition and health interventions** (dietary supplements, healthy diet, healthy eating, healthy nutrition, weight control, weight management, micronutrient supplementation diet/nutrition education, physical activity, community/home garden, and WASH, and nutrition policy)	("Health Education"[Mesh:NoExp] OR "Health Promotion"[Mesh]) OR ("Adolescent Health Services"[Mesh] OR "Preventive Health Services"[Mesh:NoExp]) OR (preventive health[tiab]) OR ("Dietary Supplements"[Mesh] OR dietary supplement*[tiab] micronutrient supplement*[tiab] OR folic acid supplement*[tiab] OR MMN[tiab] OR nutrient supplement*[tiab] OR nutritional supplement*[tiab]) OR ("Diet, Healthy"[Mesh] OR healthy diet*[tiab] OR healthy eating[tiab] OR healthy food*[tiab] OR diet education[tiab] OR dietary education[tiab] OR dietary intervention[tiab] OR healthy eating[tiab] OR healthy food*[tiab] OR healthy diet*[tiab] OR healthy nutrition*[tiab] OR nutrition counsel*[tiab] OR nutritional counsel*[tiab]) OR (nutrition education[tiab] OR nutrition intervention[tiab] OR nutritional education[tiab] OR nutritional intervention[tiab]) OR ("exercise"[MeSH] OR "exercise" [tiab] OR "physical activity"[tiab] OR fitness*[tiab] OR sport*[tiab]) OR ("nutrition policy"[MeSH] OR "nutrition"[tiab] AND "policy"[tiab] OR "nutrition policy"[tiab])	865,050<br>687,256 (2000-2023)
#3	**Adolescents**	(“Adolescent"[Mesh] OR adolescent[tiab] OR adolescents[tiab] OR adolescence[tiab] OR teen[tiab] OR teens[tiab] OR teenage*[tiab])	2,310,507<br>1,421,658 (2000-2023)
#4	**Low- and middle-income countries**	(Afghanistan*[tiab] OR Albania*[tiab] OR Algeria*[tiab] OR Samoa*[tiab] OR Angola*[tiab] OR Armenia*[tiab] OR Azerbaijan*[tiab] OR Bangladesh*[tiab] OR Bengali[tiab] OR Belarus*[tiab] OR Belize[tiab] OR Benin[tiab] OR Bhutan*[tiab] OR Bolivia*[tiab] OR Bosnia*[tiab] OR Herzegovina*[tiab] OR Botswana*[tiab] OR Brazil*[tiab] OR Bulgaria*[tiab] OR “Burkina Faso”[tiab] OR Burkinabe[tiab] OR Burundi*[tiab] OR “Cabo Verd*”[tiab] OR “Cape	1,931,426<br>1,565,369 (2000-2023)

		Verd*”[tiab] OR Cambodia*[tiab] OR Cameroon*[tiab] OR “Central African*”[tiab] OR Chad*[tiab] OR China[tiab] OR Chinese[tiab] OR Colombia*[tiab] OR Comoros[tiab] OR Congo[tiab] OR “Costa Rica*”[tiab] OR “Cote d'Ivoire”[tiab] OR “Ivory Coast”[tiab] OR Cuba[tiab] OR Cuban[tiab] OR Djibouti[tiab] OR Dominica*[tiab] OR Ecuador[tiab] OR Egypt*[tiab] OR “El Salvador*”[tiab] OR Eritrea*[tiab] OR Ethiopia*[tiab] OR Fiji*[tiab] OR Gabon*[tiab] OR Gambia*[tiab] OR Georgia*[tiab] OR Ghana*[tiab] OR Grenada*[tiab] OR Guatemala*[tiab] OR Guinea*[tiab] OR Guyan*[tiab] OR Haiti*[tiab] OR Hondura*[tiab] OR India[tiab] OR Indian*[tiab] OR Indonesia*[tiab] OR Iran*[tiab] OR Iraq*[tiab] OR Jamaica*[tiab] OR Jordan*[tiab] OR Kazakh*[tiab] OR Kenya*[tiab] OR Kiribati[tiab] OR “People's Republic of Korea”[tiab] OR “North Korea”[tiab] OR Kosovo[tiab] OR Kosovar*[tiab] OR Kyrgyz*[tiab] OR Lao[tiab] OR Laos[tiab] OR Laotian*[tiab] OR Lebanon[tiab] OR Lebanes*[tiab] OR Lesotho[tiab] OR Liberia*[tiab] OR Libya*[tiab] OR Macedonia*[tiab] OR Madagascar*[tiab] OR Malawi*[tiab] OR Malaysia*[tiab] OR Maldives[tiab] OR Mali[tiab] OR “Marshall Island*”[tiab] OR “Mexico”[MeSH] OR Mexico[tiab] OR Mexican*[tiab] OR Micronesia*[tiab] OR Moldova*[tiab] OR Mongolia*[tiab] OR Montenegr*[tiab] OR Morocc*[tiab] OR Mozambique[tiab] OR Myanmar[tiab] OR Burmese*[tiab] OR Burma[tiab] OR Namibia*[tiab] OR Nepal*[tiab] OR Nicaragua*[tiab] OR Niger*[tiab] OR Pakistan*[tiab] OR Paraguay*[tiab] OR Peru*[tiab] OR Philippin*[tiab] OR Rwanda*[tiab] OR “Sao Tome”[tiab] OR Principe[tiab] OR Senegal*[tiab] OR Serbia*[tiab] OR “Sierra Leone*”[tiab] OR “Solomon Island*”[tiab] OR Somalia*[tiab] OR “South Africa*”[tiab] OR “Sri Lanka”[tiab] OR “St Lucia”[tiab] OR “Saint Lucia”[tiab] OR “St Vincent”[tiab] OR “Saint Vincent”[tiab] OR Grenad*[tiab] OR Sudan*[tiab] OR Suriname*[tiab] OR Swaziland*[tiab] OR Eswatini*[tiab] OR Syria*[tiab] OR Tajik*[tiab] OR Tanzania*[tiab] OR Zanzibar[tiab] OR Thai*[tiab] OR Timor*[tiab] OR Togo*[tiab] OR Tonga*[tiab] OR Tunisia*[tiab] OR Turkey[tiab] OR Turkish[tiab] OR Turkmen*[tiab] OR Tuvalu*[tiab] OR Uganda*[tiab] OR Ukrain*[tiab] OR Uzbeki*[tiab] OR Vanuatu*[tiab] OR Venezuela*[tiab] OR Vietnam*[tiab] OR “Viet nam*”[tiab] OR “West Bank”[tiab] OR Gaza*[tiab] OR Palestin*[tiab] OR Yemen*[tiab] OR Zambia*[tiab] OR Zimbabw*[tiab] OR “Western Sahara”[tiab] OR Argentin*[tiab] OR Russia*[tiab] OR Maurit*[tiab] OR Palau[tiab] OR Romania*[tiab])	
	**#1 AND # 2 AND #3 AND #4**		**10,670**<br>**9,869 (2000-2023)**

No.	Concept	EMBASE search terms	Number of records (As per 14th July)
#1	**Randomized controlled trial Controlled before-after studies Quasi experimental studies**	('randomized controlled trial':af OR 'randomization'/exp OR 'crossover procedure'/exp OR 'epidemiology'/exp OR quasi experiment*:ti,ab OR quasiexperiment*:ti,ab OR 'quasi-experiment*':ti,ab)	146,384<br>131,436 (2000-2023)
#2	**Nutrition and health interventions** (dietary supplements, healthy diet, healthy eating, healthy nutrition, weight control, weight management, micronutrient supplementation diet/nutrition education, physical activity, community/home garden, and WASH, and nutrition policy)	('health education'/de OR 'health promotion'/exp) OR ('child health care'/exp OR 'preventive health service'/de) OR (preventive health:ti,ab) OR ('dietary supplement'/exp OR dietary supplement*:ti,ab micronutrient supplement*:ti,ab OR folic acid supplement*:ti,ab OR MMN:ti,ab OR nutrient supplement*:ti,ab OR nutritional supplement*:ti,ab) OR ('healthy diet'/exp OR healthy diet*:ti,ab OR healthy eating:ti,ab OR healthy food*:ti,ab OR diet education:ti,ab OR dietary education:ti,ab OR dietary intervention:ti,ab OR healthy eating:ti,ab OR healthy food*:ti,ab OR healthy diet*:ti,ab OR healthy nutrition*:ti,ab OR nutrition counsel*:ti,ab OR nutritional counsel*:ti,ab) OR (nutrition education:ti,ab OR nutrition intervention:ti,ab OR nutritional education:ti,ab OR nutritional intervention:ti,ab) OR ('exercise'/exp OR ‘exercise’:ti,ab OR ‘physical activity’:ti,ab OR fitness*:ti,ab OR sport*:ti,ab) OR ('nutrition policy'/exp OR ‘nutrition’:ti,ab AND ‘policy’:ti,ab OR ‘nutrition policy’:ti,ab)	1,421,386<br>1,168,709 (2000-2023)
#3	**Adolescents**	('adolescent'/exp OR adolescent:ti,ab OR adolescents:ti,ab OR adolescence:ti,ab OR teen:ti,ab OR teens:ti,ab OR teenage*:ti,ab)	2,031,554<br>1,398,667 (2000-2023)
#4	**Low- and middle-income countries**	(Afghanistan*:ti,ab OR Albania*:ti,ab OR Algeria*:ti,ab OR Samoa*:ti,ab OR Angola*:ti,ab OR Armenia*:ti,ab OR Azerbaijan*:ti,ab OR Bangladesh*:ti,ab OR Bengali:ti,ab OR Belarus*:ti,ab OR Belize:ti,ab OR Benin:ti,ab OR Bhutan*:ti,ab OR Bolivia*:ti,ab OR Bosnia*:ti,ab OR Herzegovina*:ti,ab OR Botswana*:ti,ab OR Brazil*:ti,ab OR Bulgaria*:ti,ab OR ‘Burkina Faso’:ti,ab OR Burkinabe:ti,ab OR Burundi*:ti,ab OR ‘Cabo Verd*’:ti,ab OR ‘Cape Verd*’:ti,ab OR Cambodia*:ti,ab OR Cameroon*:ti,ab OR ‘Central African*’:ti,ab OR Chad*:ti,ab OR China:ti,ab OR Chinese:ti,ab OR Colombia*:ti,ab OR Comoros:ti,ab OR Congo:ti,ab OR ‘Costa Rica*’:ti,ab OR ‘cote d`ivoire’:ti,ab OR ‘Ivory Coast’:ti,ab OR Cuba:ti,ab OR Cuban:ti,ab OR Djibouti:ti,ab OR Dominica*:ti,ab OR Ecuador:ti,ab OR Egypt*:ti,ab OR ‘El Salvador*’:ti,ab OR Eritrea*:ti,ab OR Ethiopia*:ti,ab OR Fiji*:ti,ab OR Gabon*:ti,ab OR Gambia*:ti,ab OR Georgia*:ti,ab OR Ghana*:ti,ab OR Grenada*:ti,ab OR Guatemala*:ti,ab OR Guinea*:ti,ab OR	2,466,552<br>2,047,567 (2000-2023)

		Guyan*:ti,ab OR Haiti*:ti,ab OR Hondura*:ti,ab OR India:ti,ab OR Indian*:ti,ab OR Indonesia*:ti,ab OR Iran*:ti,ab OR Iraq*:ti,ab OR Jamaica*:ti,ab OR Jordan*:ti,ab OR Kazakh*:ti,ab OR Kenya*:ti,ab OR Kiribati:ti,ab OR 'People`s Republic of Korea':ti,ab OR 'North Korea':ti,ab OR Kosovo:ti,ab OR Kosovar*:ti,ab OR Kyrgyz*:ti,ab OR Lao:ti,ab OR Laos:ti,ab OR Laotian*:ti,ab OR Lebanon:ti,ab OR Lebanes*:ti,ab OR Lesotho:ti,ab OR Liberia*:ti,ab OR Libya*:ti,ab OR Macedonia*:ti,ab OR Madagascar*:ti,ab OR Malawi*:ti,ab OR Malaysia*:ti,ab OR Maldives:ti,ab OR Mali:ti,ab OR 'Marshall Island*':ti,ab OR 'Mexico'/exp OR Mexico:ti,ab OR Mexican*:ti,ab OR Micronesia*:ti,ab OR Moldova*:ti,ab OR Mongolia*:ti,ab OR Montenegr*:ti,ab OR Morocc*:ti,ab OR Mozambique:ti,ab OR Myanmar:ti,ab OR Burmese*:ti,ab OR Burma:ti,ab OR Namibia*:ti,ab OR Nepal*:ti,ab OR Nicaragua*:ti,ab OR Niger*:ti,ab OR Pakistan*:ti,ab OR Paraguay*:ti,ab OR Peru*:ti,ab OR Philippin*:ti,ab OR Rwanda*:ti,ab OR 'Sao Tome":ti,ab OR Principe:ti,ab OR Senegal*:ti,ab OR Serbia*:ti,ab OR 'Sierra Leone*':ti,ab OR 'Solomon Island*':ti,ab OR Somalia*:ti,ab OR 'South Africa*':ti,ab OR 'Sri Lanka':ti,ab OR 'St Lucia':ti,ab OR 'Saint Lucia':ti,ab OR 'St Vincent':ti,ab OR 'Saint Vincent':ti,ab OR Grenad*:ti,ab OR Sudan*:ti,ab OR Suriname*:ti,ab OR Swaziland*:ti,ab OR Eswatini*:ti,ab OR Syria*:ti,ab OR Tajik*:ti,ab OR Tanzania*:ti,ab OR Zanzibar:ti,ab OR Thai*:ti,ab OR Timor*:ti,ab OR Togo*:ti,ab OR Tonga*:ti,ab OR Tunisia*:ti,ab OR Turkey:ti,ab OR Turkish:ti,ab OR Turkmen*:ti,ab OR Tuvalu*:ti,ab OR Uganda*:ti,ab OR Ukrain*:ti,ab OR Uzbeki*:ti,ab OR Vanuatu*:ti,ab OR Venezuela*:ti,ab OR Vietnam*:ti,ab OR 'Viet nam*':ti,ab OR 'West Bank':ti,ab OR Gaza*:ti,ab OR Palestin*:ti,ab OR Yemen*:ti,ab OR Zambia*:ti,ab OR Zimbabw*:ti,ab OR 'Western Sahara':ti,ab OR Argentin*:ti,ab OR Russia*:ti,ab OR Maurit*:ti,ab OR Palau:ti,ab OR Romania*:ti,ab)	
	**#1 AND # 2 AND #3 AND #4**		**17,874**<br>**16,942 (2000-2023)**

No.	Concept	Cochrane search terms	Number of records (As of July 14th 2023)
#1	**Randomized controlled trial**<br>**Controlled before-after studies**<br>**Quasi experimental studies**	#1 "randomized controlled trial"<br>#2 [mh "random allocation"]<br>#3 {OR #1-#2}<br>#4 [mh "cross-over studies"]<br>#5 [mh "Controlled Before-After Studies"]<br>#6 {OR #4-#5}<br>#7 (quasi NEXT experiment*):ti,ab<br>#8 quasiexperiment*:ti,ab<br>#9 (quasi-experiment*):ti,ab<br>#10 {OR #7-#9}	702,362<br>664,394<br>(2000-2023)
#2	**Nutrition and health interventions**<br>(Dietary supplements, healthy diet, healthy eating, healthy nutrition, overweight, anaemia, obesity, weight control, weight management, micronutrient supplementation diet/nutrition education, school meal, physical activity, school garden, and WASH, and nutrition policy)	#11 [mh ^"health education"] OR [mh "health promotion"]<br>#12 [mh "Adolescent Health Services"] OR [mh ^"Preventive Health Services"]<br>#13 [mh "Adolescent Health Services"]<br>#14 [mh ^"Preventive Health Services"]<br>#15 [mh "Dietary Supplements"] OR dietary supplement*:ti,ab OR micronutrient supplement*:ti,ab OR folic acid supplement*:ti,ab OR folic acid supplement*:ti,ab OR MMN:ti,ab OR nutrient supplement*:ti,ab OR nutritional supplement*:ti,ab<br>#16 [mh "Diet, Healthy"] OR healthy diet*:ti,ab OR healthy eating:ti,ab OR healthy food*:ti,ab OR diet education:ti,ab OR dietary education:ti,ab OR dietary intervention:ti,ab OR healthy nutrition*:ti,ab OR nutrition counsel*:ti,ab OR nutritional counsel*:ti,ab<br>#17 nutrition education:ti,ab OR nutrition intervention:ti,ab OR nutritional education:ti,ab OR nutritional intervention:ti,ab<br>#18 [mh exercise] OR exercise:ti,ab OR physical activity:ti,ab OR fitness*:ti,ab OR sport*:ti,ab<br>#19 [mh "nutrition policy"] OR nutrition:ti,ab AND policy:ti,ab OR "nutrition policy":ti,ab<br>#20 {OR #11-#19}	237.951<br>222,406<br>(2000-2023)
#3	**Adolescents**	#21 [mh Adolescent] OR adolescent:ti,ab OR adolescents:ti,ab OR adolescence:ti,ab<br>#22 teen:ti,ab OR teens:ti,ab OR teenage*:ti,ab<br>#23 {OR #21-#22}	146,504<br>115,839<br>(2000-2023)
#4	**Low- and middle-income countries**	#24 (Afghanistan*):ti,ab OR (Albania*):ti,ab OR (Algeria*):ti,ab OR (Samoa*):ti,ab OR (Angola*):ti,ab<br>#25 (Armenia*):ti,ab OR (Azerbaijan*):ti,ab OR (Bangladesh*):ti,ab OR (Bengali):ti,ab OR (Belarus*):ti,ab<br>#26 (Belize):ti,ab OR (Benin):ti,ab OR (Bhutan*):ti,ab OR (Bolivia*):ti,ab OR (Bosnia*):ti,ab<br>#27 (Herzegovina*):ti,ab OR (Botswana*):ti,ab OR (Brazil*):ti,ab OR (Bulgaria*):ti,ab OR ("Burkina Faso"):ti,ab OR Burkinabe:ti,ab<br>#28 Burundi*:ti,ab OR (Cabo NEXT Verd*):ti,ab OR (Cape NEXT Verd*):ti,ab OR Cambodia*:ti,ab OR Cameroon*:ti,ab<br>#29 (Central African*):ti,ab OR (Chad*):ti,ab OR (China):ti,ab OR (Chinese):ti,ab OR (Colombia*):ti,ab	128,522<br>124,301<br>(2000-2023)

		#30 (Comoros):ti,ab OR (Congo):ti,ab OR ("Cook Islands"):ti,ab OR (Costa NEXT Rica*):ti,ab OR ("Cote d'Ivoire"):ti,ab<br>#31 ("Ivory Coast"):ti,ab OR (Cuba):ti,ab OR (Cuban):ti,ab OR (Djibouti):ti,ab OR (Dominica*):ti,ab<br>#32 (Ecuador):ti,ab OR (Egypt):ti,ab OR (El NEXT Salvador*):ti,ab OR (Eritrea*):ti,ab OR (Ethiopia*):ti,ab<br>#33 (Fiji*):ti,ab OR (Gabon*):ti,ab OR (Gambia*):ti,ab OR (Georgia*):ti,ab<br>#34 (Ghana*):ti,ab OR (Grenada*):ti,ab OR (Guadeloupe):ti,ab OR (Guatemala*):ti,ab<br>#35 (Guinea*):ti,ab OR (Guyan*):ti,ab OR (Haiti*):ti,ab OR (Hondura*):ti,ab OR (India):ti,ab<br>#36 (Indian*):ti,ab OR (Indonesia*):ti,ab OR (Iran*):ti,ab OR (Iraq*):ti,ab OR (Jamaica*):ti,ab<br>#37 (Jordan*):ti,ab OR (Kazakh*):ti,ab OR (Kenya*):ti,ab OR (Kiribati):ti,ab OR ("People's Republic of Korea"):ti,ab<br>#38 ("North Korea"):ti,ab OR (Kosovo):ti,ab OR (Kosovar*):ti,ab OR (Kyrgyz*):ti,ab OR (Lao):ti,ab<br>#39 (Laos):ti,ab OR (Laotian*):ti,ab OR (Lebanon):ti,ab OR (Lebanes*):ti,ab OR (Lesotho):ti,ab<br>#40 (Liberia*):ti,ab OR (Libya*):ti,ab OR (Macedonia*):ti,ab OR (Madagascar*):ti,ab OR (Malawi*):ti,ab<br>#41 (Malaysia*):ti,ab OR (Maldives):ti,ab OR (Mali):ti,ab OR (Marshall NEXT Island*):ti,ab<br>#42 [mh Mexico] OR Mexico:ti,ab OR (Mexican*):ti,ab<br>#43 (Micronesia*):ti,ab OR (Moldova*):ti,ab OR (Mongolia*):ti,ab OR (Montenegr*):ti,ab OR (Morocc*):ti,ab<br>#44 (Mozambique):ti,ab OR (Myanmar):ti,ab OR (Burmese*):ti,ab OR (Burma):ti,ab OR (Namibia*):ti,ab<br>#45 Nepal*:ti,ab OR Nicaragua*:ti,ab OR Niger*:ti,ab OR Pakistan*:ti,ab OR Paraguay*:ti,ab<br>#46 Peru*:ti,ab OR Philippin*:ti,ab OR Rwanda*:ti,ab OR "Sao Tome":ti,ab OR Principe:ti,ab OR Senegal*:ti,ab OR Serbia*:ti,ab<br>#47 (Sierra NEXT Leone*):ti,ab OR (Solomon NEXT Island*):ti,ab OR Somalia*:ti,ab OR (South NEXT Africa*):ti,ab OR "Sri Lanka":ti,ab<br>#48 "St Lucia":ti,ab OR "Saint Lucia":ti,ab OR "St Vincent":ti,ab OR "Saint Vincent":ti,ab OR Grenad*:ti,ab<br>#49 Sudan*:ti,ab OR Suriname*:ti,ab OR Swaziland*:ti,ab OR Eswatini*:ti,ab OR Syria*:ti,ab OR Tajik*:ti,ab OR Tanzania*:ti,ab<br>#50 Zanzibar:ti,ab OR Thai*:ti,ab OR Timor*:ti,ab OR Togo*:ti,ab OR Tonga*:ti,ab OR Tunisia*:ti,ab<br>#51 Turkey:ti,ab OR Turkish:ti,ab OR Turkmen*:ti,ab OR Tuvalu*:ti,ab OR Uganda*:ti,ab OR Ukrain*:ti,ab<br>#52 Uzbeki*:ti,ab OR Vanuatu*:ti,ab OR Venezuela*:ti,ab OR Vietnam*:ti,ab OR Viet nam*:ti,ab<br>#53 Palestin*:ti,ab OR Yemen*:ti,ab OR Zambia*:ti,ab OR Zimbabw*:ti,ab OR "Western Sahara":ti,ab<br>#54 Argentin*:ti,ab OR Russia*:ti,ab OR Maurit*:ti,ab OR Palau:ti,ab OR Romania*:ti,ab<br>#55 {OR #24-#54}	
	**#1 AND # 2 AND #3 AND #4**	(#3 OR #6 OR #10) AND #20 AND #23 AND #55	**1,504**<br>**1,504**<br>**(2000-2023)**

Supplementary File 2. Sample of extraction form

	Publication details						
Sr. No	Title	Journal/ source	Calendar year of publication	first author	corresponding author	contact author email	funding agency

Study Methods								
Calendar year of study	Country	Study type	Study design	Target population (age, gender, socioeconomic status, etc.)	Sample size	Inclusion criteria	Exclusion criteria	Analysis methods

Intervention												
Intervention name	Timing of intervention	Duration of intervention	Guiding theory/ framework	Intervention topics	Intervention components/ activities	Frequency and duration of intervention activities	Intervention delivery mechanism	Intervention delivery agents	Selection, training, and supervision of delivery agents	Intervention coverage	Control group intervention

Outcome							
Outcome 1	Timepoint for Outcome 1 assessment	Outcome 1 Methods of measurement	Outcome 1 measure (units and tools)	Outcome *X*	Timepoint for outcome X assessment	Outcome X methods of measurement	Outcome X measure (units and tools)

Results			
**Quantitative findings**	**Qualitative findings**	**Theory to explain the success**	**Theory to explain the failure**
