# Supplementary material for: Community-based interventions targeting multiple forms of malnutrition among adolescents in low-income and middle-income countries: protocol for a scoping review
Source: BMJ Open. 2024 Mar 28;14(3):e078969. doi: 10.1136/bmjopen-2023-078969 (PMC10982731; doi:10.1136/bmjopen-2023-078969)
Supplement: Author's manuscript [file bmjopen-2023-078969.draft_revisions.pdf]

BMJ Open is committed to open peer review. As part of this commitment we make the peer review history of every article we publish publicly available.

When an article is published we post the peer reviewers' comments and the authors' responses online. We also post the versions of the paper that were used during peer review. These are the versions that the peer review comments apply to.

The versions of the paper that follow are the versions that were submitted during the peer review process. They are not the versions of record or the final published versions. They should not be cited or distributed as the published version of this manuscript.

BMJ Open is an open access journal and the full, final, typeset and author-corrected version of record of the manuscript is available on our site with no access controls, subscription charges or pay-per-view fees (http://bmjopen.bmj.com).

If you have any questions on BMJ Open's open peer review process please email info.bmjopen@bmj.com

BMJ Open

# Community-based interventions targeting multiple forms of malnutrition among adolescents in low- and middle-income countries: protocol for a scoping review

| | |
|---|---|
| Journal: | *BMJ Open* |
| Manuscript ID | bmjopen-2023-078969 |
| Article Type: | Protocol |
| Date Submitted by the Author: | 17-Aug-2023 |
| Complete List of Authors: | Kurniawan, Adi Lukas; Heidelberg University, Heidelberg Institute of Global Health<br>Ranisavljev, Marijana; University of Novi Sad, Faculty of Sport and Physical Education<br>Partap, Uttara; Harvard University T H Chan School of Public Health, Department of Global Health and Population<br>Shinde, Sachin ; Harvard University T H Chan School of Public Health, Department of Global Health and Population; Central for Inquiry into Mental Health<br>Ostojic, Sergej; University of Novi Sad, Faculty of Medicine; University of Agder, Department of Nutrition and Public Health<br>Mkwanazi, Ntombizodumo; University of KwaZulu-Natal, Research Division<br>Alangea, Deda Ogum; University of Ghana, Department of Population, Family, and Reproductive Health<br>Neumann, Christine; Heidelberg University, Heidelberg Institute of Global Health<br>Liu, Shuyan; Charité Universitätsmedizin Berlin, Department of Psychiatry and Psychotherapy<br>Bärnighausen, Till ; Heidelberg University, Heidelberg Institute of Global Health; Africa Health Research Institute<br>Fawzi, Wafaie.; Harvard University T H Chan School of Public Health, Department of Global Health and Population; Harvard University T H Chan School of Public Health, Department of Nutrition<br>Collaborators, ARISE-NUTRINT; Heidelberg University, Heidelberg Institute of Global Health; Harvard University T H Chan School of Public Health |
| Keywords: | Adolescent, NUTRITION & DIETETICS, Health Education, Systematic Review, Anaemia < HAEMATOLOGY |
| | |

SCHOLARONE™
Manuscripts

# Community-based interventions targeting multiple forms of malnutrition among adolescents in low- and middle-income countries: protocol for a scoping review

Adi Lukas Kurniawan [1], Marijana Ranisavljev [2], Uttara Partap [3], Sachin Shinde [3,4,*], Sergej Ostojic [5,6], Ntombizodumo Mkwanazi [7], Deda Ogum Alangea [8], Christine Neumann [1], Shuyan Liu [9], Till Bärnighausen [1,10], Wafaie W Fawzi [3,11,12], ARISE-NUTRINT collaborators [13].

[1]Heidelberg Institute of Global Health, Heidelberg University Hospital, Heidelberg, Germany. Email: lukas.kurniawan@uni-heidelberg.de (ALK); till.baernighausen@uni-heidelberg.de (TB); christine.neumann@uni-heidelberg.de (CN)

[2]Faculty of Sport and Physical Education, University of Novi Sad, Vojvodina, Serbia. Email: marijanaranisavljev@gmail.com (MR)

[3]Department of Global Health and Population, Harvard T. H. Chan School of Public Health, Boston, Massachusetts, USA. Email: mina@hsph.harvard.edu (WWF); upartap@hsph.harvard.edu (UP)

[4]Center for Inquiry into Mental Health, Pune, Maharashtra, India.

[5]Faculty of Medicine, University of Novi Sad, Novi Sad, Serbia. Email: sergej.ostojic@chess.edu.rs (SO)

[6]Department of Nutrition and Public Health, University of Agder, Kristiansand, Norway.

[7]Research Division, University of KwaZulu-Natal, Durban, South Africa. Email: MkwanaziN2@ukzn.ac.za (NM)

[8]Department of Population, Family, and Reproductive Health, School of Public Health, University of Ghana, Accra, Ghana. Email: DOgumAlangea@ug.edu.gh (DOA)

[9]Department of Psychiatry and Neurosciences, Charité Berlin University Medicine, Berlin, Germany. Email: shuyan.liu@charite.de (SL)

[10]Africa Health Research Institute, Durban, South Africa.

[11]Department of Epidemiology, Harvard T. H. Chan School of Public Health, Boston, Massachusetts, USA.

[12]Department of Nutrition, Harvard T. H. Chan School of Public Health, Boston, Massachusetts, USA.

[13]ARISE-NUTRINT collaborators:

For peer review only

Michael Laxy, Professorship of Public Health and Prevention, Technical University of Munich, Germany.

Jacob Burns, Professorship of Public Health and Prevention, Technical University of Munich, Germany.

Sara Pedron, Professorship of Public Health and Prevention, Technical University of Munich, Germany.

Mary Mwanyika Sando, Africa Academy for Public Health, Tanzania.

Ayoade Oduola, University of Ibadan Research Foundation, Nigeria.

Mosa Moshabela, University of KwaZulu-Natal, South Africa.

Ali Sié, National Institute of Public Health, Nouna Health Research Center, Burkina Faso.

David Guwatudde, School of Public Health, Makerere University, Uganda.

Yemane Berhane, Addis Continental Institute of Public Health, Ethiopia.

Adom Manu, Department of Population, Family, and Reproductive Health, University of Ghana, Ghana.

Jan A.C. Hontelez, Erasmus Universitair Medisch Centrum, The Netherlands.

Magda Rosenmöller, Center for Research in Healthcare Innovation Management, IESE Business School, Spain.

Irene Brandt, Heidelberg Institute of Global Health, Heidelberg University Hospital, Germany.

Ina Danquah, Heidelberg Institute of Global Health, Heidelberg University Hospital, Germany.

Matthias Kern, Heidelberg Institute of Global Health, Heidelberg University Hospital, Germany.

Joy Mauti, Heidelberg Institute of Global Health, Heidelberg University Hospital, Germany.

Shannon McMahon, Heidelberg Institute of Global Health, Heidelberg University Hospital, Germany.

Japhet Killewo, Muhimbili University of Health and Allied Sciences, Tanzania.

Hanna Y. Berhane, Department of Nutrition and Behavioral Sciences, Addis Continental Institute of Public Health, Ethiopia.

Amani Tinkasimile, Africa Academy for Public Health, Tanzania.

Mashavu Yussuf, Africa Academy for Public Health, Tanzania.

Innocent Yusufu, Africa Academy for Public Health, Tanzania.

Laetitia Paumard, Center for Research in Healthcare Innovation Management, IESE Business School, Spain.

For peer review only

Millogo Ourohiré, National Institute of Public Health, Nouna Health Research Center, Burkina Faso.

Erick Agure, Heidelberg Institute of Global Health, Heidelberg University Hospital, Germany.

Jabulani Ncayiyana, College of Health Sciences, University of KwaZulu-Natal, South Africa.

Bruno Sunguya, School of Public Health and Social Sciences, Muhimbili University of Health and Allied Sciences, Tanzania.

Tiwatayo Lasebikan, Center for Research in Healthcare Innovation Management, IESE Business School, Spain.

Marina Taonda, National Institute of Public Health, Nouna Health Research Center, Burkina Faso.

Sylvain Somé, National Institute of Public Health, Nouna Health Research Center, Burkina Faso.

Clarisse Dah, National Institute of Public Health, Nouna Health Research Center, Burkina Faso.

Katian Napon, National Institute of Public Health, Nouna Health Research Center, Burkina Faso.

Moussa Ouédraogo, National Institute of Public Health, Nouna Health Research Center, Burkina Faso.

Elisabetta Ferrero, Department of Global Health and Population, Harvard T. H. Chan School of Public Health, USA.

*Correspondence: Sachin Shinde, Department of Global Health and Population, Harvard T.H. Chan School of Public Health, 3rd Floor, 90 Smith St. Boston, MA 02120, USA. Email: sshinde@hsph.harvard.edu

**Word count:** 3010 (not including title page, abstract, references, tables, or acknowledgement)

For peer review only

**Abstract**

**Background:** Adolescent malnutrition is a significant public health challenge in low- and middle-income countries (LMICs), with long-term consequences for health and development. Community-based interventions have the potential to address multiple forms of malnutrition and improve the health outcomes of adolescents. However, there is a lack of comprehensive understanding regarding such interventions' content, implementation, and effectiveness. This scoping review aims to synthesize evidence on community-based interventions targeting multiple forms of malnutrition among adolescents in LMICs and describe their effects on nutrition and health.

**Methods and analysis:** A comprehensive search strategy will be implemented in multiple databases including MEDLINE (through PubMed), Embase, and CENTRAL (through Cochrane Library), covering the period from January 1, 2000, to July 14, 2023. The inclusion criteria encompass randomized controlled trials and quasi-experimental studies focusing on adolescents aged 10-19 years. Various types of interventions, such as micronutrient supplementation, nutrition education, feeding interventions, physical activity, and community environment interventions, will be considered. Two reviewers will perform data extraction independently, and, where relevant, risk of bias assessment will be conducted using standard Cochrane risk-of-bias tools. The findings will be synthesized and presented using the Synthesis Without Meta-analysis (SWiM) guidelines for scoping reviews.

**Ethics and dissemination:** The scope of this scoping review is restricted to publicly accessible databases that do not require prior ethical approval for access. The findings of this review will be shared through publications in peer-reviewed journals, and presentations at international and regional conferences and stakeholder meetings in LMICs.

**Scoping review registration:** The final protocol was registered prospectively with the Open Science Framework on July 19, 2023 (https://osf.io/t2d78).

For peer review only

99 **Article Summary**

100 Strengths and limitations of this study

101 - This scoping review will clearly describe the available evidence on community-based interventions that address the multiple forms of malnutrition among adolescents in LMICs.
103 - Grey literature sources such as government reports and organization websites will be also included.
104 - Alongside consideration of the characteristics of documented interventions, we will also consider measures of impact.
106 - Where measures of impact are available, there will be a quality assessment of the included studies.
107 - The proposed search strategy will be conducted only in three electronic databases.

For peer review only

**Introduction**

The current global adolescent population surpasses 1.2 billion individuals, with approximately 90 percent of them residing in low- and middle-income countries (LMICs) (1). Moreover, when considering regional trends, it is expected that the proportion of young people aged 10 to 24 residing in Sub-Saharan Africa will experience substantial growth, rising from 245 million in 2015 to 605 million by 2050 (2). In contrast, the Asia and Pacific region is expected to undergo the most significant decrease, declining from 718 million in 2015 to 619 million by 2050 (2). These regional variations emphasize the unique challenges and opportunities faced by different regions in terms of future demographic and health challenges, which will require distinct solutions.

Adolescence is a period of rapid physical growth, cognitive development, socio-emotional development, and cultural development, all of which are strongly influenced by an individual's socioeconomic, cultural, and physical environments (3). Nutrition plays a crucial role in improving health and development during this critical stage in life, bringing intergenerational benefits. After the first 1000 days of life, adolescence is assumed to offer a second opportunity for correcting nutritional deficiencies and insufficient growth since childhood (4).

Low- and middle-income countries (LMICs) are experiencing a rapid nutrition transition among adolescents, accompanied on the one hand by stunting, thinness, anemia, and other micronutrient deficiencies, and on the other hand by an increasing burden of obesity and non-communicable diseases (5). Malnutrition was the leading cause of disability-adjusted life years (DALYs) among the 10-14 age group in 2019, followed by iron deficiency anemia among adolescents aged 10-19 (6). The consumption of diverse and healthy diets by adolescents from LMICs is declining, while the consumption of processed and calorie-rich foods is on the rise, contributing to rising obesity rates (7). Furthermore, food insecurity has been aggravated in vulnerable populations including adolescents in LMICs because of the COVID-19 pandemic, political instability, and recurring climate crises in the form of flooding and droughts.

For peer review only

Several systematic reviews indicate that micronutrient supplementation is effective in addressing nutritional deficiencies (8). Iron supplementation can reduce anemia in adolescents; periconceptional folic acid supplementation among adolescent girls can reduce neural tube defects; and adolescent girls who consume high amounts of calcium (≥1 g daily) have lower rates of preeclampsia, preterm birth, or neonatal hospitalization (9). There is limited evidence that protein-energy supplements are effective for adolescents.

The burden of malnutrition may be reduced by several nutrition-sensitive interventions, including nutrition education, dietary interventions, physical activity, and food environment interventions (10). Several systematic reviews suggest promising but modest results from discrete nutrition-sensitive interventions aimed at addressing malnutrition in schools (11-13). These single-domain interventions, however, target either undernutrition or overnutrition and operate in *silos*. There is increased interest in addressing health and nutrition behaviors through integrated interventions, generally called "double-duty actions", targeting multiple forms of malnutrition and nutrition-related non-communicable diseases (14). An essential element of this concept is that tackling one form of malnutrition should not prevent addressing another. There is promising evidence that integrated interventions can improve the nutritional status of school-going children and adolescents (10).

Nevertheless, there are several gaps in understanding adolescent nutrition in LMICs. Currently, nutrition-specific, and nutrition-sensitive interventions tend to focus on school-going adolescents, and little is known about their effects on other vulnerable groups of adolescents, such as out-of-school adolescents, migrant adolescents, and HIV-positive adolescents. Another important gap is that most of the school-based interventions target overlapping age groups and there is little known regarding the age-appropriate intervention strategies and delivery mechanisms as well as the specific impact on the adolescent population. Moreover, most of these school-based interventions are delivered by schoolteachers, community health workers, school nurses, or peers in classroom-based settings or during school hours. Despite the importance of nutrition-sensitive and nutrition-specific interventions for the health of communities, little evidence

For peer review only

exists about their form and function using community platforms. Therefore, the purpose of this scoping review is to comprehensively review the literature to describe community-based interventions that address the multiple forms of malnutrition such as obese, overweight, underweight, wasting, stunting, anemia, and micronutrient deficiencies affecting adolescents in LMICs and describe the effects of these interventions on nutrition and health.

## Methods

### Data sources, search terms and search strategy

We will search MEDLINE (through PubMed), Embase, and CENTRAL (through the Cochrane Library). All databases will be searched for eligible studies from January 1, 2000, through July 14, 2023. We will identify potentially relevant published studies using the combination of medical subject headings (MeSH) and text words denoting nutrition-specific and nutrition-sensitive interventions. We will also examine references and bibliographies of included studies to identify additional sources of information. This search of studies will be supplemented by reviewing ClinicalTrials.gov and organizational websites such as the World Health Organization (WHO), World Bank, United Nations Children's Fund (UNICEF), and United Nations Population Fund (UNFPA). When possible, reports written in languages other than English will be translated by colleagues who are native speakers of those languages. No study will be considered if it cannot be adequately translated.

We will use the PICO model (Table 1) to guide our search strategy, but we will not be restricted by outcomes to maintain a broad search. The search will use indexing terms, including MeSH terms, keywords, and free text words. First, a broad search strategy (e.g., type of study [randomized controlled trials, quasi-experiments, or controlled before-after studies] AND intervention domain [e.g., nutrition education] AND population [adolescents] AND setting [low- and middle-income countries]) will be performed in PubMed. We will confirm the sensitivity of the search strategy by identifying several sentinel articles. The PubMed strategy, provided in **Supplementary File 1**, will be adapted to suit other databases. We will document the

For peer review only - http://bmjopen.bmj.com/site/about/guidelines.xhtml

following details for each search: databases searched, date of search, search strategy (i.e., subject headings and keywords, including if terms are expanded, truncated, and how they are combined), filters used, and the number of records retrieved. Additionally, a source will be provided for each publication identified through manual search (i.e., journal name, website, conference proceedings, etc.).

**Table 1 Eligibility criteria for the systematic review in PICO format**

| **Item** | **Inclusion criteria** | **Exclusion criteria** |
|---|---|---|
| Population | Studies involving adolescents (10–19 years old) in the community setting in low- and middle-income countries | Studies involving adolescents (10–19 years old) but interventions applied exclusively in the school setting |
| Interventions, approaches or expo-sures of interests | Studies involving one or more of the following interventions: nutrient supplementation interventions including vitamin and nutrient supplementation, deworming, complementary feeding, nutrition education, physical education, promoting healthy diets and/or physical activity, nutrition policies, community/home garden, water, sanitation and hygiene interventions, community environment interventions, and structural interventions such as sweetened beverage tax, soda tax, and sugary drink tax | Interventions targeted towards individuals with specific medical conditions such as treatments intended for underweight, overweight, or obese adolescents |

For peer review only

| | | |
|---|---|---|
| Comparison | Studies that compared the intervention with any relevant control group including comparisons with no intervention, regular nutrition education and/or physical education, or any other intervention in the community setting | Not applicable |
| Outcomes | Body-Mass Index (BMI) z score, anemia, change in anthropometry (e.g., height and weight status, BMI, height-for-age z scores, weight-for-age z scores, weight-for-height z scores, skin-fold thickness measures, stunting, underweight, wasting, body mass index, overweight, obesity, waist-to-height ratio, and central obesity), hemoglobin level in the blood, micronutrient deficiencies, knowledge of diet and nutrition, dietary intake (i.e. amount and frequency), dietary diversity, diet quality, physical activity, sedentary behaviors, nutrition literacy, and nutrition fluency. | Not applicable |

For peer review only

| | | |
|---|---|---|
| Study design | Randomized controlled trials, quasi-experimental studies including controlled before-after studies | Non-randomized trials including controlled before-after studies that did not account for baseline differences, observational studies including cohort, case-control, and cross-sectional designs, and editorial commentaries, opinions, and review articles |

**Eligibility**

The inclusion and exclusion criteria for this scoping review are listed below.

***Inclusion criteria***

We will include the following studies.

- We will include randomized controlled trials (RCT), with the intervention randomized to individuals or in clusters (including clubs, groups, communities, villages, homes, etc.), and quasi-experimental studies including controlled before-after studies that have reported interventions to address any form of adolescent malnutrition when compared to a control group.
- Studies involving adolescent boys and/or girls aged 10-19 years.
- Studies conducted in LMICs—as defined by the World Bank in the year 2023 (15).
- Studies involving interventions for one or more of the following: micronutrient supplementation, feeding interventions, nutrition education, physical education, interventions to promote healthy diets, interventions promoting physical activity, community and/or home gardens, food and nutrition policies, community environment interventions, water sanitation and hygiene (WASH) interventions, and structural interventions such as taxation of sweetened-sugary drinks.

For peer review only - http://bmjopen.bmj.com/site/about/guidelines.xhtml

- The control (comparison) in each included study can be participants who did not receive any intervention or received standard care, received standard health/nutrition education, or any other intervention in the community setting.
- We will include published articles as well as unpublished and grey literature and will include ongoing studies where preliminary findings are available.
- We will not place any restrictions on the language, sample size, or duration of the intervention.

***Exclusion criteria***

We will not consider the following studies.

- Non-RCTs that are not quasi-experimental studies with comparator groups and controlled before-after studies that did not account for the baseline differences between the study arms.
- Observational studies such as cohort, case-control, and cross-sectional designs.
- Editorials, commentaries, opinions, and review articles. However, we will use review articles to identify additional original articles.
- Clinical treatments/interventions targeted towards individuals with specific medical conditions such as programs intended for underweight, overweight, obese, or anemic adolescents.

**Data management**

EndNote X9 (Clarivate Analytics, Pennsylvania, United States) or a similar citation management software will be used to store the records retrieved from electronic databases. The records will also be imported into Covidence (Veritas Health Innovation, Melbourne, Australia), an Internet-based systematic review management program. Detection and removal of duplicates will be performed by EndNote (or similar software) and by Covidence.

**Selection of studies**

Using Covidence, we will screen titles, abstracts, and full texts. First, two reviewers will independently assess all search results (i.e., titles and abstracts) and exclude irrelevant studies based on inclusion and exclusion criteria. Next, two reviewers will carry out the full-text screening based on the same inclusion/exclusion criteria. The reviewers will discuss and resolve any difference of opinion or, if necessary, seek a third reviewer's opinion for resolving differences. A study flow diagram stating the specific reasons for exclusion will be maintained following the PRISMA for Scoping Review statement (PRISMA-ScR) (16).

**Data extraction**

Two reviewers will independently extract and enter data from studies included in the review. We will develop and test an extraction form on five randomly selected studies. We will extract the following information.

- Study details including the title, authors (first author and corresponding author), the corresponding author's contact information, journal (or source for unpublished reports), calendar year of publication, calendar year of intervention, country, and source of funding.
- Study methods including objectives and/or research questions, type of study, investigation strategies, settings, sample size, and sample characteristics (e.g., age, sex, socioeconomic status
- Intervention strategy including target population, delivery platform and providers (including selection, training, supervision, support, and incentivization), types of nutrition and other interventions (including content, conceptual framework and/or theoretical underpinnings, timing, duration, and dosage or frequency), and comparator/control.
- Outcomes assessed and details of the measures used.
- Findings including the coverage of services, facilitators and barriers to intervention delivery and uptake, effectiveness findings with point estimates and measures of variance (standard errors, 95%

For peer review only

confidence intervals, or *p*-values), and any other key findings related to the scoping review questions.

We will contact the corresponding author via email if there is missing or inconsistent information. We will contact the author two times at most. The available data will be analyzed and any gaps due to missing data will be discussed if the data issue cannot be resolved after contacting the authors.

**Risk of bias assessment**

For the assessment of the risk of bias in the selected studies, we will use the Cochrane Collaboration's revised tool for assessing the risk of bias in randomized trials (RoB 2) (17). Two reviewers will independently evaluate methodological quality. Any uncertainties or disagreements will be resolved by discussion or by a third reviewer, whenever needed. The tool is a domain-based evaluation, in which critical assessments for risk of bias are made separately for various domains, including the randomization process, deviation from intended interventions, missing outcome data, measurement of the outcome, and selective outcome reporting. The risk of bias in clustered trials will be similarly assessed using the risk of bias 2 for cluster-randomized trials (RoB 2 CRT) (18). Additionally, we will use the Risk of Bias in Non-randomized Studies of Interventions (ROBINS-I) tool (19), to assess the risk of bias for controlled before-after studies and non-randomized controlled trials.

**Synthesis of evidence**

All included studies will be systematically synthesized in the text and a table following the SWiM guidelines (Synthesis Without Meta-analysis) (20). In this synthesis, we will describe how many sources of evidence were screened, assessed for eligibility, and included in the review, along with reasons for exclusion at each stage. Our presentation of included sources of evidence will include summary characteristics and citations, as well as a critical appraisal, if applicable. Studies will be grouped based on methods and interventions, standardized outcomes metrics, synthesis methods, criteria used to prioritize

For peer review only

results for summary, reporting of results, the certainty of results, heterogeneity in effects, as well as barriers and facilitators to delivering the interventions will be discussed. For continuous outcomes, effect estimates will be expressed as mean differences (with 95% confidence intervals) between the intervention group and the control group; for dichotomous outcomes, effect estimates will be expressed as risk ratios, rate ratios, hazard ratios, or odds ratios (all with a 95% confidence interval). Additionally, we will discuss the limitations of the review process and provide an interpretation of the results concerning the objectives of the review, as well as possible implications or next steps. We will follow the PRISMA Extension for Scoping Reviews (PRISMA-ScR) checklist and guidelines to ensure a robust and replicable process (16).

**Registration and reporting**

The final protocol was registered prospectively with the Open Science Framework (https://osf.io/t2d78) on July 19, 2023, based on the PRISMA Extension for Scoping Reviews (PRISMA-ScR) (16). In the event of protocol amendments, the date of each amendment will be accompanied by a description of each change and the rationale on the Open Science Forum.

**Discussion**

The fight against malnutrition is one of the greatest global health challenges, influenced by economic growth, urbanization, and globalization, as well as shifts in the quality and quantity of human diets. For the period 2016-2025, the United Nations Decade of Action on Nutrition was launched, calling for specific coordinated actions through cross-cutting and coherent policies, programs, and initiatives to address all forms of malnutrition (21). The global community has transitioned from a predominant focus on eliminating severe and acute undernutrition through the Millennium Development Goals (MDGs) to a broader focus on nutrition through the Sustainable Development Goals (SDGs). It is an opportunity for integrated action to address all forms of malnutrition and non-communicable diseases. According to Sustainable Development 2030, tracking indicators are also needed to meet targets, specifically for the Global Strategy for Women's,

Children's, and Adolescents' Health (22). In this review, we will describe and synthesize community-based interventions targeting the many forms of malnutrition among adolescents aged 10-19 from LMICs.

There are a variety of interventions that could be implemented in schools, including the most common and longest-standing one— school feeding (10, 23). Schools provide a platform for interventions that target high-risk groups beyond food, including adolescent girls receiving weekly iron and folic acid supplements (WIFA), nutrition and physical education, promoting a healthy diet and active lifestyle, school gardens and/or farms, creating positive school environments, and water and sanitation interventions (10). While school enrollment has increased significantly since 2000 across the globe and is acknowledged as a central platform for improving nutrition among school-going children and adolescents, around 37% of secondary school-age children in sub-Saharan Africa do not attend school and many of those who do attend are irregular students (24). There is, therefore, a need for new approaches that will improve nutrition literacy, ensure food security, and address specific nutrition challenges among out-of-school adolescents. Consequently, community-based nutrition-specific and nutrition-sensitive interventions targeting multiple forms of malnutrition outcomes of adolescents are needed to synthesize the evidence. In order to improve the nutritional status of adolescents, it is imperative to engage adolescents, families, and communities, and to allow health, education, nutrition, social protection, and other agencies to work together synergistically.

Community-based interventions provide opportunities to engage with a range of adolescent groups and collaborate with various local institutions. Communities can provide interventions and support strategies to improve local food environments and food choices, ranging from non-governmental organizations and religious groups to local businesses, self-help groups, and sports or music clubs (25). Many of the most effective obesity interventions consider a specific social context and are delivered in community settings in high-income countries. For example, in one US study, mentoring from African American community members increased the impact of nutrition interventions on African American young people (26). It is also possible to target social norms and the environmental context of nutritional choices through community

For peer review only

interventions. With regard to social influences more broadly, adolescents today have access to a much wider range of social networks than their predecessors. As a result of social media and information technology, both social and commercial influences in adolescence have evolved (27). Social media has sometimes been associated with harmful nutrition outcomes—for example, reinforcing weight-related stigma and normalizing unhealthy body image—but it has also helped young people connect with people outside their immediate communities and transformed social opportunities for less mobile people. Despite their enormous potential, there is limited literature evaluating the impact of community-based interventions in LMICs.

Considering the recommendations of the Lancet Commission on Adolescent Health (3), the WHO's Global Accelerated Action for Adolescent Health (28), and the Child and Adolescent Health Volume of Disease Control Priorities (29) that advocate the integration of health and nutrition, we anticipate the results of this review will serve to advance the application of these recommendations. Using the results of this review, future programs can be developed to address the immediate and growing needs of adolescents in community settings. In addition, future programs can include approaches to connecting such interventions with schools, families, and the broader community. In addition, these findings can help policymakers, researchers, practitioners, government agencies, and non-governmental organizations develop and implement interventions to improve adolescents' integrated health and address malnutrition and other health outcomes in LMICs.

For peer review only

**Ethics and dissemination**

This study is a scoping review that does not require ethics approval because it involves a methodical presentation of available resources. The protocol aims to provide an overview of the broad literature on community-based interventions targeting multiple forms of malnutrition among adolescents in LMICs. Additionally, the submitted review will help identify effective interventions, determine gaps and disparities

among interventions, and provide insight for policymakers to develop and design as well as implement future programs.

**Author Contributors**

SS conceived the idea, developed the methods, and wrote the first draft of the manuscript. ALK, MR, and UP contributed to the methods and supported the drafting and editing of the manuscript. ALK and MR contributed meaningfully to the design of the search strategy. SO, NM, DOA, CN, SL, TB, WWF, and ARISE-NUTRINT study collaborators supervised and reviewed the protocol. All authors revised and approved the final manuscript.

**Funding**

This study was funded by the European Union Horizon 2022. Views and opinions expressed are however those of the author(s) only and do not necessarily reflect those of the European Union. Neither the European Union nor the granting authority can be held responsible for them.

**Competing interests**

None declared.

**Patient consent for publication**

Not required

**Provenance and peer review**

Not commissioned, externally peer-reviewed

**References**

1. United Nations Children's Fund. Progress for Children: A Report Card on Adolescents No. 10. 2012. Available at www.unicef.org/publications/files/Progress_for_Children_-_No._10_EN_04272012.pdf.
2. United Nations. Department of Economic and Social Affairs, Population Division. World population prospects, the 2012 revision. Available at: https://population.un.org/wpp/

For peer review only

3. Patton GC, Sawyer SM, Santelli JS, et al. Our future: a Lancet commission on adolescent health and well-being. Lancet 2016;387:2423-78.

4. Patton GC, Olsson C, Skirbekk V, et al. Adolescence and the next generation. Nature 2018;554: 458–66.

5. Canavan CR, Fawzi WW. Addressing knowledge gaps in adolescent nutrition: Towards advancing public health and sustainable development. Curr Dev Nutr 2019;3:nzz062.

6. World Health Organization. Global health estimates: leading causes of DALYs. Available at: https://www.who.int/data/gho/data/themes/mortality-and-global-health-estimates/global-health-estimates-leading-causes-of-dalys

7. Norris SA, Frongillo EA, Black MM, et al. Nutrition in adolescent growth and development. Lancet 2022;299:172-84.

8. Hargreaves D, Mates E, Menon P, et al. Strategies and interventions for healthy adolescent growth, nutrition and development. Lancet 2022;299:198-210.

9. Roche ML, Samson KLI, Green TJ, et al. Perspective: Weekly Iron and Folic Acid Supplementation (WIFAS): A critical review and rationale for inclusion in the essential medicines list to accelerate anemia and neural tube defects reduction. Adv Nutr. 2021;12:334-42.

For peer review only

10. Shinde S, Wang D, Moulton GE, et al. School-based health and nutrition interventions addressing double burden of malnutrition and educational outcomes of adolescents in low- and middle-income countries: A systematic review. Matern Child Nutr 2023:e13437. doi: 10.1111/mcn.13437

11. Kyere P, Veerman JL, Lee P, et al. Effectiveness of school-based nutrition interventions in sub-Saharan Africa: a systematic review. Public Health Nutr 2020;23:2626-36.

12. Van Cauwenberghe E, Maes L, Spittaels H, et al. Effectiveness of school-based interventions in Europe to promote healthy nutrition in children and adolescents: systematic review of published and 'grey' literature. Br J Nutr 2010;103:781-97.

13. Pongutta S, Ajetunmobi O, Davey C, et al. Impacts of school nutrition interventions on the nutritional status of school-aged children in Asia: A systematic review and meta-analysis. Nutrients. 2022;14:589. doi: 10.3390/nu14030589.

14. Hawkes C, Ruel M, Salm L, et al. Double-duty actions: seizing programme and policy opportunities to address malnutrition in all its forms. Lancet 2019;395:142-55.

15. World Bank designated developing countries 2023. World bank. Available at: https://www.endocrine.org/membership/join/developing-country-list

16. Tricco AC, Lillie E, Zarin W, et al. PRISMA Extension for Scoping Reviews (PRISMA-ScR): Checklist and Explanation. Ann Intern Med 2018;169:467-73.

17. Sterne JAC, Savović J, Page MJ, et al. RoB 2: a revised tool for assessing the risk of bias in randomized trials. BMJ 2019;366:l4898.

For peer review only

18. Eldridge S, Campbell M, Cambell M, et al. Revised Cochrane risk of bias tool for randomized trials (RoB 2.0) additional considerations for cluster randomized trials. Available at: https://www.unisa.edu.au/contentassets/72bf75606a2b4abcaf7f17404af374ad/rob2-0_cluster_parallel_guidance.pdf

19. Sterne JA, Hernán MA, Reeves BC, et al. ROBINS-I: a tool for assessing the risk of bias in non-randomized studies of interventions. BMJ 2016;355: i4919.

20. Campbell M, McKenzie JE, Sowden A, et al. Synthesis without meta-analysis (SWiM) in systematic reviews: reporting guideline. BMJ 2020;368:l6890.

21. United Nations. United Nations Decade of Action on Nutrition 2016-2025. www.un.org/nutrition.

22. Kuruvilla S, Bustreo F, Kuo T, et al. The Global strategy for women's children's and adolescents' health (2016–2030): a roadmap based on evidence and country experience. Bull World Health Organ 2016;94:398–400.

23. Wang D, Shinde S, Young T, et al. Impacts of school feeding on educational and health outcomes of school-age children and adolescents in low- and middle-income countries: A systematic review and meta-analysis. J Glob Health 2021;11:04051.

24. The World Bank Adolescents out of school (% of lower secondary school age). Available at: https://data.worldbank.org/indicator/SE.SEC.UNER.LO.ZS

25. Hargreaves D, Mates E, Menon P, et al. Strategies and interventions for healthy adolescent growth, nutrition, and development. Lancet 2022;399:198-210.

For peer review only

438 26. Lofton S, Julion WA, McNaughton DB, et al. A systematic review of literature on culturally adapted obesity prevention interventions for African American youth. J Sch Nur 2016;32:32-46.

440 27. Chau MM, Burgermaster M, Mamykina L. The use of social media in nutrition interventions for adolescents and young adults-A systematic review. Int J Med Inform 2018;120:77-91.

442 28. World Health Organization. Global Accelerated Action for the Health of Adolescents (AA-HA!): guidance to support country implementation. Geneva: World Health Organization; 2017.

444 29. Lassi Z, Moin A, Bhutta Z. Nutrition in middle childhood and adolescence. (in Eds) Bundy D, de'Silva N, Horton S, Hamison DT, Patton GC. Child and adolescent health development. Disease Control Priorities 3rd Edition. Washington DC: World Bank, 2017.

For peer review only

**Supplementary file 1: PubMed search strategy**

| No. | Concept | PubMed search terms |
|---|---|---|
| #1 | **Randomized controlled trial Controlled before-after studies Quasi-experimental studies** | ("randomized controlled trial"[pt] OR "random allocation"[mesh] OR "cross-over studies"[mesh] OR "Controlled Before-After Studies"[Mesh] OR quasi experiment*[tiab] OR quasiexperiment*[tiab] OR "quasi-experiment*"[tiab]) |
| #2 | **Nutrition and health interventions** (dietary supplements, healthy diet, healthy eating, healthy nutrition, weight control, weight management, micronutrient supplementation diet/nutrition education, physical activity, community/home garden, WASH, and nutrition policy) | ("Health Education"[Mesh:NoExp] OR "Health Promotion"[Mesh]) OR ("Adolescent Health Services"[Mesh] OR "Preventive Health Services"[Mesh:NoExp]) OR (preventive health[tiab]) OR ("Dietary Supplements"[Mesh] OR dietary supplement*[tiab] micronutrient supplement*[tiab] OR folic acid supplement*[tiab] OR MMN[tiab] OR nutrient supplement*[tiab] OR nutritional supplement*[tiab]) OR ("Diet, Healthy"[Mesh] OR healthy diet*[tiab] OR healthy eating[tiab] OR healthy food*[tiab] OR diet education[tiab] OR dietary education[tiab] OR dietary intervention[tiab] OR healthy eating[tiab] OR healthy food*[tiab] OR healthy diet*[tiab] OR healthy nutrition*[tiab] OR nutrition counsel*[tiab] OR nutritional counsel*[tiab]) OR (nutrition education[tiab] OR nutrition intervention[tiab] OR nutritional education[tiab] OR nutritional intervention[tiab]) OR ("exercise"[MeSH] OR "exercise" [tiab] OR "physical activity"[tiab] OR fitness*[tiab] OR sport*[tiab]) OR ("nutrition policy"[MeSH] OR "nutrition"[tiab] AND "policy"[tiab] OR "nutrition policy"[tiab]) |
| #3 | **Adolescents** | ("Adolescent"[Mesh] OR adolescent[tiab] OR adolescents[tiab] OR adolescence[tiab] OR teen[tiab] OR teens[tiab] OR teenage*[tiab]) |
| #4 | **Low- and middle-income countries** | (Afghanistan*[tiab] OR Albania*[tiab] OR Algeria*[tiab] OR Samoa*[tiab] OR Angola*[tiab] OR Armenia*[tiab] OR Azerbaijan*[tiab] OR Bangladesh*[tiab] OR Bengali[tiab] OR Belarus*[tiab] OR Belize[tiab] OR Benin[tiab] OR Bhutan*[tiab] OR Bolivia*[tiab] OR Bosnia*[tiab] OR Herzegovina*[tiab] OR Botswana*[tiab] OR Brazil*[tiab] OR Bulgaria*[tiab] OR "Burkina Faso"[tiab] OR Burkinabe[tiab] OR Burundi*[tiab] OR "Cabo Verd*"[tiab] OR "Cape Verd*"[tiab] OR Cambodia*[tiab] OR Cameroon*[tiab] OR "Central African*"[tiab] OR Chad*[tiab] OR China[tiab] OR Chinese[tiab] OR Colombia*[tiab] OR Comoros[tiab] OR Congo[tiab] OR "Costa Rica*"[tiab] OR "Cote d'Ivoire"[tiab] OR "Ivory Coast"[tiab] OR Cuba[tiab] OR Cuban[tiab] OR Djibouti[tiab] OR Dominica*[tiab] OR Ecuador[tiab] OR Egypt*[tiab] OR "El Salvador*"[tiab] OR Eritrea*[tiab] OR Ethiopia*[tiab] OR Fiji*[tiab] OR Gabon*[tiab] OR Gambia*[tiab] OR Georgia*[tiab] OR Ghana*[tiab] OR Grenada*[tiab] OR Guatemala*[tiab] OR Guinea*[tiab] OR Guyan*[tiab] OR Haiti*[tiab] OR Hondura*[tiab] OR India[tiab] OR Indian*[tiab] OR Indonesia*[tiab] OR Iran*[tiab] OR Iraq*[tiab] OR Jamaica*[tiab] OR Jordan*[tiab] OR Kazakh*[tiab] OR Kenya*[tiab] OR Kiribati[tiab] OR "People's Republic of Korea"[tiab] OR "North Korea"[tiab] OR Kosovo[tiab] OR Kosovar*[tiab] OR Kyrgyz*[tiab] OR Lao[tiab] OR Laos[tiab] OR Laotian*[tiab] OR Lebanon[tiab] OR Lebanes*[tiab] OR Lesotho[tiab] OR Liberia*[tiab] OR |

| | | |
|---|---|---|
| | | Libya*[tiab] OR Macedonia*[tiab] OR Madagascar*[tiab] OR Malawi*[tiab] OR Malaysia*[tiab] OR Maldives[tiab] OR Mali[tiab] OR "Marshall Island*"[tiab] OR "Mexico"[MeSH] OR Mexico[tiab] OR Mexican*[tiab] OR Micronesia*[tiab] OR Moldova*[tiab] OR Mongolia*[tiab] OR Montenegr*[tiab] OR Morocc*[tiab] OR Mozambique[tiab] OR Myanmar[tiab] OR Burmese*[tiab] OR Burma[tiab] OR Namibia*[tiab] OR Nepal*[tiab] OR Nicaragua*[tiab] OR Niger*[tiab] OR Pakistan*[tiab] OR Paraguay*[tiab] OR Peru*[tiab] OR Philippin*[tiab] OR Rwanda*[tiab] OR "Sao Tome"[tiab] OR Principe[tiab] OR Senegal*[tiab] OR Serbia*[tiab] OR "Sierra Leone*"[tiab] OR "Solomon Island*"[tiab] OR Somalia*[tiab] OR "South Africa*"[tiab] OR "Sri Lanka"[tiab] OR "St Lucia"[tiab] OR "Saint Lucia"[tiab] OR "St Vincent"[tiab] OR "Saint Vincent"[tiab] OR Grenad*[tiab] OR Sudan*[tiab] OR Suriname*[tiab] OR Swaziland*[tiab] OR Eswatini*[tiab] OR Syria*[tiab] OR Tajik*[tiab] OR Tanzania*[tiab] OR Zanzibar[tiab] OR Thai*[tiab] OR Timor*[tiab] OR Togo*[tiab] OR Tonga*[tiab] OR Tunisia*[tiab] OR Turkey[tiab] OR Turkish[tiab] OR Turkmen*[tiab] OR Tuvalu*[tiab] OR Uganda*[tiab] OR Ukrain*[tiab] OR Uzbeki*[tiab] OR Vanuatu*[tiab] OR Venezuela*[tiab] OR Vietnam*[tiab] OR "Viet nam*"[tiab] OR "West Bank"[tiab] OR Gaza*[tiab] OR Palestin*[tiab] OR Yemen*[tiab] OR Zambia*[tiab] OR Zimbabw*[tiab] OR "Western Sahara"[tiab] OR Argentin*[tiab] OR Russia*[tiab] OR Maurit*[tiab] OR Palau[tiab] OR Romania*[tiab]) |

For peer review only

BMJ Open

# Community-based interventions targeting multiple forms of malnutrition among adolescents in low- and middle-income countries: protocol for a scoping review

| | |
|---|---|
| Journal: | *BMJ Open* |
| Manuscript ID | bmjopen-2023-078969.R1 |
| Article Type: | Protocol |
| Date Submitted by the Author: | 23-Feb-2024 |
| Complete List of Authors: | Kurniawan, Adi Lukas; Heidelberg University, Heidelberg Institute of Global Health<br>Ranisavljev, Marijana; University of Novi Sad, Faculty of Sport and Physical Education<br>Partap, Uttara; Harvard University T H Chan School of Public Health, Department of Global Health and Population<br>Shinde, Sachin ; Harvard University T H Chan School of Public Health, Department of Global Health and Population; Central for Inquiry into Mental Health<br>Ferrero, Elisabetta; Harvard University T H Chan School of Public Health<br>Ostojic, Sergej; University of Novi Sad, Faculty of Medicine; University of Agder, Department of Nutrition and Public Health<br>Mkwanazi, Ntombizodumo; University of KwaZulu-Natal, Research Division<br>Alangea, Deda Ogum; University of Ghana, Department of Population, Family, and Reproductive Health<br>Neumann, Christine; Heidelberg University, Heidelberg Institute of Global Health<br>Liu, Shuyan; Charité Universitätsmedizin Berlin, Department of Psychiatry and Psychotherapy<br>Bärnighausen, Till ; Heidelberg University, Heidelberg Institute of Global Health; Africa Health Research Institute<br>Fawzi, Wafaie.; Harvard University T H Chan School of Public Health, Department of Global Health and Population; Harvard University T H Chan School of Public Health, Department of Nutrition<br>Collaborators, ARISE-NUTRINT; Heidelberg University, Heidelberg Institute of Global Health; Harvard University T H Chan School of Public Health |
| <b>Primary Subject Heading</b>: | Nutrition and metabolism |
| Secondary Subject Heading: | Global health, Public health, Research methods |
| Keywords: | Adolescent, NUTRITION & DIETETICS, Health Education, Systematic Review, Anaemia < HAEMATOLOGY |
| | |

# Community-based interventions targeting multiple forms of malnutrition among adolescents in low- and middle-income countries: protocol for a scoping review

Adi Lukas Kurniawan [1], Marijana Ranisavljev [2], Uttara Partap [3], Sachin Shinde [3,4,*], Elisabetta Ferrero [3], Sergej Ostojic [5,6], Ntombizodumo Mkwanazi [7], Deda Ogum Alangea [8], Christine Neumann [1], Shuyan Liu [9], Till Bärnighausen [1,10], Wafaie W Fawzi [3,11,12], ARISE-NUTRINT collaborators [13].

[1]Heidelberg Institute of Global Health, Heidelberg University Hospital, Heidelberg, Germany. Email: lukas.kurniawan@uni-heidelberg.de (ALK); till.baernighausen@uni-heidelberg.de (TB); christine.neumann@uni-heidelberg.de (CN)

[2]Faculty of Sport and Physical Education, University of Novi Sad, Vojvodina, Serbia. Email: marijanaranisavljev@gmail.com (MR)

[3]Department of Global Health and Population, Harvard T. H. Chan School of Public Health, Boston, Massachusetts, USA. Email: mina@hsph.harvard.edu (WWF); upartap@hsph.harvard.edu (UP)

[4]Center for Inquiry into Mental Health, Pune, Maharashtra, India.

[5]Faculty of Medicine, University of Novi Sad, Novi Sad, Serbia. Email: sergej.ostojic@chess.edu.rs (SO)

[6]Department of Nutrition and Public Health, University of Agder, Kristiansand, Norway.

[7]Research Division, University of KwaZulu-Natal, Durban, South Africa. Email: MkwanaziN2@ukzn.ac.za (NM)

[8]Department of Population, Family, and Reproductive Health, School of Public Health, University of Ghana, Accra, Ghana. Email: DOgumAlangea@ug.edu.gh (DOA)

[9]Department of Psychiatry and Neurosciences, Charité Berlin University Medicine, Berlin, Germany. Email: shuyan.liu@charite.de (SL)

[10]Africa Health Research Institute, Durban, South Africa.

[11]Department of Epidemiology, Harvard T. H. Chan School of Public Health, Boston, Massachusetts, USA.

[12]Department of Nutrition, Harvard T. H. Chan School of Public Health, Boston, Massachusetts, USA.

[13]ARISE-NUTRINT collaborators:

For peer review only



For peer review only - http://bmjopen.bmj.com/site/about/guidelines.xhtml

Michael Laxy, Professorship of Public Health and Prevention, Technical University of Munich, Germany.

Jacob Burns, Professorship of Public Health and Prevention, Technical University of Munich, Germany.

Sara Pedron, Professorship of Public Health and Prevention, Technical University of Munich, Germany.

Mary Mwanyika Sando, Africa Academy for Public Health, Tanzania.

Ayoade Oduola, University of Ibadan Research Foundation, Nigeria.

Mosa Moshabela, University of KwaZulu-Natal, South Africa.

Ali Sié, National Institute of Public Health, Nouna Health Research Center, Burkina Faso.

David Guwatudde, School of Public Health, Makerere University, Uganda.

Yemane Berhane, Addis Continental Institute of Public Health, Ethiopia.

Adom Manu, Department of Population, Family, and Reproductive Health, University of Ghana, Ghana.

Jan A.C. Hontelez, Erasmus Universitair Medisch Centrum, The Netherlands.

Magda Rosenmöller, Center for Research in Healthcare Innovation Management, IESE Business School, Spain.

Irene Brandt, Heidelberg Institute of Global Health, Heidelberg University Hospital, Germany.

Ina Danquah, Heidelberg Institute of Global Health, Heidelberg University Hospital, Germany.

Matthias Kern, Heidelberg Institute of Global Health, Heidelberg University Hospital, Germany.

Joy Mauti, Heidelberg Institute of Global Health, Heidelberg University Hospital, Germany.

Shannon McMahon, Heidelberg Institute of Global Health, Heidelberg University Hospital, Germany.

Japhet Killewo, Muhimbili University of Health and Allied Sciences, Tanzania.

Hanna Y. Berhane, Department of Nutrition and Behavioral Sciences, Addis Continental Institute of Public Health, Ethiopia.

Amani Tinkasimile, Africa Academy for Public Health, Tanzania.

Mashavu Yussuf, Africa Academy for Public Health, Tanzania.

Innocent Yusufu, Africa Academy for Public Health, Tanzania.

Laetitia Paumard, Center for Research in Healthcare Innovation Management, IESE Business School, Spain.

For peer review only



For peer review only - http://bmjopen.bmj.com/site/about/guidelines.xhtml

Millogo Ourohiré, National Institute of Public Health, Nouna Health Research Center, Burkina Faso.

Erick Agure, Heidelberg Institute of Global Health, Heidelberg University Hospital, Germany.

Jabulani Ncayiyana, College of Health Sciences, University of KwaZulu-Natal, South Africa.

Bruno Sunguya, School of Public Health and Social Sciences, Muhimbili University of Health and Allied Sciences, Tanzania.

Tiwatayo Lasebikan, Center for Research in Healthcare Innovation Management, IESE Business School, Spain.

Marina Taonda, National Institute of Public Health, Nouna Health Research Center, Burkina Faso.

Sylvain Somé, National Institute of Public Health, Nouna Health Research Center, Burkina Faso.

Clarisse Dah, National Institute of Public Health, Nouna Health Research Center, Burkina Faso.

Katian Napon, National Institute of Public Health, Nouna Health Research Center, Burkina Faso.

Moussa Ouédraogo, National Institute of Public Health, Nouna Health Research Center, Burkina Faso.

*Correspondence: Sachin Shinde, Department of Global Health and Population, Harvard T.H. Chan School of Public Health, 3rd Floor, 90 Smith St. Boston, MA 02120, USA. Email: sshinde@hsph.harvard.edu

**Word count:** 2581 (not including title page, abstract, references, tables, or acknowledgement)

For peer review only



For peer review only - http://bmjopen.bmj.com/site/about/guidelines.xhtml

**Abstract**

**Background:** Adolescent malnutrition is a significant public health challenge in low- and middle-income countries (LMICs), with long-term consequences for health and development. Community-based interventions have the potential to address multiple forms of malnutrition and improve the health outcomes of adolescents. However, there is a limited understanding of the content, implementation, and effectiveness of these interventions. This scoping review aims to synthesize evidence on community-based interventions targeting multiple forms of malnutrition among adolescents in LMICs and describe their effects on nutrition and health.

**Methods and analysis:** A comprehensive search strategy will be implemented in multiple databases including MEDLINE (through PubMed), Embase, CENTRAL (through Cochrane Library), and grey literature, covering the period from January 1, 2000, to July 14, 2023. We will follow the Participants, Concept, and Context (PCC) model to design the search strategy. The inclusion criteria encompass randomized controlled trials and quasi-experimental studies focusing on adolescents aged 10-19 years. Various types of interventions, such as micronutrient supplementation, nutrition education, feeding interventions, physical activity, and community environment interventions, will be considered. Two reviewers will perform data extraction independently, and, where relevant, risk of bias assessment will be conducted using standard Cochrane risk-of-bias tools. We will follow the PRISMA Extension for Scoping Reviews checklist while reporting results.

**Ethics and dissemination:** The scope of this scoping review is restricted to publicly accessible databases that do not require prior ethical approval for access. The findings of this review will be shared through publications in peer-reviewed journals, and presentations at international and regional conferences and stakeholder meetings in LMICs.

**Scoping review registration:** The final protocol was registered prospectively with the Open Science Framework on July 19, 2023 (https://osf.io/t2d78).

For peer review only



For peer review only - http://bmjopen.bmj.com/site/about/guidelines.xhtml

**Article Summary**

Strengths and limitations of this study

- A comprehensive examination of over 20 years of published data will be conducted.
- Grey literature sources such as government reports and organization websites will also be included.
- There will be a quality assessment of the included quantitative studies.
- The proposed search strategy will be conducted only in three electronic databases.

For peer review only



For peer review only - http://bmjopen.bmj.com/site/about/guidelines.xhtml

**Introduction**

The current global adolescent population surpasses 1.2 billion individuals, with approximately 90 percent of them residing in low- and middle-income countries (LMICs) (1). Moreover, when considering regional trends, it is expected that the proportion of young people aged 10 to 24 residing in Sub-Saharan Africa will experience substantial growth, rising from 245 million in 2015 to 605 million by 2050 (2). In contrast, the Asia and Pacific region is expected to undergo the most significant decrease, declining from 718 million in 2015 to 619 million by 2050 (2). These regional variations emphasize the unique challenges and opportunities faced by different regions in terms of future demographic and health challenges, which will require distinct solutions.

Adolescence is a period of rapid physical growth, cognitive development, socio-emotional development, and cultural development, all of which are strongly influenced by an individual's socioeconomic, cultural, and physical environments (3). Nutrition plays a crucial role in improving health and development during this critical stage in life, bringing intergenerational benefits. After the first 1000 days of life, adolescence is assumed to offer a second window of opportunity for correcting nutritional deficiencies and insufficient growth since childhood (4).

LMICs are experiencing a rapid nutrition transition among adolescents, accompanied on the one hand by stunting, thinness, anemia, and other micronutrient deficiencies, and on the other hand by an increasing burden of obesity and non-communicable diseases (5). Malnutrition was the leading cause of disability-adjusted life years (DALYs) among the 10-14 age group in 2019, followed by iron deficiency anemia among adolescents aged 10-19 (6). The consumption of diverse and healthy diets by adolescents from LMICs is declining, while the consumption of processed and calorie-rich foods is on the rise, contributing to rising obesity rates (7). Furthermore, food insecurity has been aggravated in vulnerable populations including adolescents in LMICs because of the COVID-19 pandemic, political instability, and recurring climate crises in the form of flooding and droughts (8).



For peer review only - http://bmjopen.bmj.com/site/about/guidelines.xhtml

Several systematic reviews indicate that micronutrient supplementation is effective in addressing nutritional deficiencies (9,10). Iron supplementation can reduce anemia in adolescents; periconceptional folic acid supplementation among adolescent girls can reduce neural tube defects; and adolescent girls who consume high amounts of calcium (≥1 g daily) have lower rates of preeclampsia, preterm birth, or neonatal hospitalization (11). There is limited evidence that protein-energy supplements are effective for adolescents (10).

The burden of malnutrition may be reduced by several nutrition-sensitive interventions, including nutrition education, dietary interventions, physical activity, and food environment interventions (12). Several systematic reviews suggest promising but modest results from discrete nutrition-sensitive interventions aimed at addressing malnutrition in schools (13-15). These single-domain interventions, however, target either undernutrition or overnutrition and operate in *silos*. There is increased interest in addressing health and nutrition behaviors through integrated interventions, generally called "double-duty actions", targeting multiple forms of malnutrition and nutrition-related non-communicable diseases (16). An essential element of this concept is that tackling one form of malnutrition should not prevent addressing another. There is promising evidence that integrated interventions can improve the nutritional status of school-going children and adolescents (12).

Nevertheless, there are several gaps in understanding adolescent nutrition in LMICs. Currently, nutrition-specific, and nutrition-sensitive interventions tend to focus on school-going adolescents, and little is known about their effects on other vulnerable groups of adolescents, such as out-of-school adolescents, migrant adolescents, and HIV-positive adolescents. Another important gap is that most of the school-based interventions target overlapping age groups and there is little known regarding the age-appropriate intervention strategies and delivery mechanisms as well as the specific impact on the adolescent population. Moreover, most of these school-based interventions are delivered by schoolteachers, community health workers, school nurses, or peers in classroom-based settings or during school hours. Despite the importance



For peer review only - http://bmjopen.bmj.com/site/about/guidelines.xhtml

of nutrition-sensitive and nutrition-specific interventions for the health of communities, little evidence exists about their form and function using community platforms.

The purpose of this scoping review is to comprehensively review the literature to describe community-based interventions that address the multiple forms of malnutrition such as obesity, overweight, underweight, wasting, stunting, anemia, and micronutrient deficiencies affecting adolescents in LMICs, and describe the effects of these interventions on nutrition and health. We decided to conduct a scoping review as our primary aim was to summarize the overview of the evidence on community-based interventions for adolescents in LMICs, rather than to pursue a specific clinical or epidemiological question related to these or provide evidence to directly inform policy or practice (17). In the context of this review, community-based interventions refer to any interventions carried out in community settings other than schools, to improve the health among adolescents. Examples include interventions implemented through community youth centers, clubs, or religious centers. By excluding school-community interventions, which have been thoroughly explored in the literature, we can concentrate on interventions that are less common, less understood, and less easy to implement, but that have the potential to reach the most vulnerable groups of adolescents.

## Methods

### Data sources, search terms, and search strategy

As part of our primary strategy, we will search MEDLINE (through PubMed), Embase, and CENTRAL (through the Cochrane Library). All databases will be searched for eligible studies from January 1, 2000, through July 14, 2023. We will identify potentially relevant published studies using the combination of medical subject headings (MeSH) and text words denoting nutrition-specific and nutrition-sensitive interventions. We will also examine references and bibliographies of included studies to identify additional sources of information. This search of studies will be supplemented by reviewing ClinicalTrials.gov and organizational websites such as the World Health Organization (WHO), World Bank, United Nations



For peer review only - http://bmjopen.bmj.com/site/about/guidelines.xhtml

Children's Fund (UNICEF), and United Nations Population Fund (UNFPA). When possible, reports written in languages other than English will be translated by colleagues who are native speakers of those languages. No study will be considered if it cannot be adequately translated.

We will use the Participants, Concept, and Context (PCC) model (Table 1) to guide our search strategy. The search will use indexing terms, including MeSH terms, keywords, and free text words. First, a broad search strategy (e.g., type of study [randomized controlled trials, quasi-experiments, or controlled before-after studies] AND intervention domain [e.g., nutrition education] AND population [adolescents] AND setting [low- and middle-income countries]) will be performed in PubMed. We will confirm the sensitivity of the search strategy by identifying several sentinel articles. The PubMed strategy, provided in **Supplementary File 1**, will be adapted to suit other databases. We will document the following details for each search: databases searched, date of search, search strategy (i.e., subject headings and keywords, including if terms are expanded, truncated, and how they are combined), filters used, and the number of records retrieved. Additionally, a source will be provided for each publication identified through manual search (i.e., journal name, website, conference proceedings, etc.).

**Table 1. Eligibility criteria for the scoping review**

| Item | Inclusion criteria | Exclusion criteria |
| --- | --- | --- |
| Participants | Studies involving adolescents (10–19 years old) | Studies involving children <10 years of age or adults (>19 years of age) |
| Concept | Studies involving one or more of the following interventions: nutrient supplementation interventions including vitamin and nutrient supplementation, deworming, | Interventions targeted towards individuals with specific medical conditions such as treatments intended for underweight, overweight, or obese adolescents |



For peer review only - http://bmjopen.bmj.com/site/about/guidelines.xhtml

| | | |
|---|---|---|
| | complementary feeding, nutrition education, physical education, promoting healthy diets and/or physical activity, nutrition policies, community/home garden, water, sanitation and hygiene interventions, community environment interventions, and structural interventions such as sweetened beverage tax, soda tax, and sugary drink tax.<br><br>Studies that compared the intervention with any relevant control group including comparisons with no intervention, regular nutrition education and/or physical education, or any other intervention in the community setting | |
| Context | Community settings in low- and middle-income countries | Interventions applied exclusively in the school setting |
| Types of sources | Randomized controlled trials, quasi-experimental studies including controlled before-after studies | Non-randomized trials including controlled before-after studies that did not account for baseline differences, observational studies including cohort, case-control, and cross- |



For peer review only - http://bmjopen.bmj.com/site/about/guidelines.xhtml

| | | |
|---|---|---|
| | | sectional designs, and editorial commentaries, opinions, and review articles |

**Eligibility**

The inclusion and exclusion criteria for this scoping review are listed below.

***Inclusion criteria***

We will include the following studies.

- Randomized controlled trials (RCT), with the intervention randomized to individuals or in clusters (including clubs, groups, communities, villages, homes, etc.), and quasi-experimental studies including controlled before-after studies that have reported interventions to address any form of adolescent malnutrition when compared to a control group.
- Studies involving adolescent boys and/or girls aged 10-19 years, based on the WHO definition of adolescents (18).
- Studies conducted in LMICs—as defined by the World Bank in the year 2023 (19).
- Studies involving interventions for one or more of the following: micronutrient supplementation, feeding interventions, nutrition education, physical education, interventions to promote healthy diets, interventions promoting physical activity, community and/or home gardens, food and nutrition policies, community environment interventions, water sanitation and hygiene (WASH) interventions, and structural interventions such as taxation of sweetened-sugary drinks.
- The control (comparison) in each included study can be participants who did not receive any intervention or received standard care, received standard health/nutrition education, or any other intervention in the community setting.
- Published articles as well as unpublished and grey literature and will include ongoing studies where preliminary findings are available.
- We will not place any restrictions on the language, sample size, or duration of the intervention.



For peer review only - http://bmjopen.bmj.com/site/about/guidelines.xhtml

***Exclusion criteria***

We will not consider the following studies.

- Non-RCTs that are not quasi-experimental studies with comparator groups and controlled before-after studies that did not account for the baseline differences between the study arms.
- Observational studies such as cohort, case-control, and cross-sectional designs.
- Editorials, commentaries, opinions, and review articles. However, we will use review articles to identify additional original articles.
- Studies that were conducted in the school setting and clinical interventions targeted individuals with specific medical conditions such as programs intended for underweight, overweight, obese, or anemic adolescents.

**Data management**

The records will be imported into Covidence (Veritas Health Innovation, Melbourne, Australia), an Internet-based systematic review management program. Detection and removal of duplicates, title and abstract screening, and full-text screening will be performed by using Covidence.

**Selection of studies**

Using Covidence, we will screen titles, abstracts, and full texts. First, two reviewers will independently assess all search results (i.e., titles and abstracts) and exclude irrelevant studies based on inclusion and exclusion criteria. Next, two reviewers will carry out the full-text screening based on the same inclusion/exclusion criteria. The reviewers will discuss and resolve any difference of opinion or, if necessary, seek a third reviewer's opinion for resolving differences. A study flow diagram stating the specific reasons for exclusion will be maintained following the PRISMA for Scoping Review statement (PRISMA-ScR) (20).



For peer review only - http://bmjopen.bmj.com/site/about/guidelines.xhtml

**Data extraction**

Two reviewers will independently extract and enter data from studies included in the review. We will develop and test an extraction form on five randomly selected studies. We will extract the following information.

- Study details including the title, authors (first author and corresponding author), the corresponding author's contact information, journal (or source for unpublished reports), calendar year of publication, calendar year of intervention, country, and source of funding.
- Study methods including objectives and/or research questions, type of study, investigation strategies, settings, sample size, and sample characteristics (e.g., age, sex, socioeconomic status
- Intervention strategy including target population, delivery platform and providers (including selection, training, supervision, support, and incentivization), types of nutrition and other interventions (including content, conceptual framework and/or theoretical underpinnings, timing, duration, and dosage or frequency), and comparator/control.
- Outcomes assessed and details of the measures used.
- Findings including the coverage of services, facilitators and barriers to intervention delivery and uptake, effectiveness findings with point estimates and measures of variance (standard errors, 95% confidence intervals, or *p*-values), and any other key findings related to the scoping review questions.

We will contact the corresponding author via email if there is missing or inconsistent information. We will contact the author two times at most. The available data will be analyzed and any gaps due to missing data will be discussed if the data issue cannot be resolved after contacting the authors. The extraction form template was provided in **Supplementary File 2**.

**Risk of bias assessment**



For peer review only - http://bmjopen.bmj.com/site/about/guidelines.xhtml

As scoping reviews are exploratory in nature, risk of bias assessments are not typically required as part of the guidelines for scoping reviews (17). However, we plan to assess the risk of bias among studies with an available quantitative measure as a preliminary way of contextualizing the reported measures of impact on the outcomes reported. For the assessment of the risk of bias in the selected studies, we will use the Cochrane Collaboration's revised tool for assessing the risk of bias in randomized trials (RoB 2) (21). Two reviewers will independently evaluate methodological quality. Any uncertainties or disagreements will be resolved by discussion or by a third reviewer, whenever needed. The tool is a domain-based evaluation, in which critical assessments for risk of bias are made separately for various domains, including the randomization process, deviation from intended interventions, missing outcome data, measurement of the outcome, and selective outcome reporting. The risk of bias in clustered trials will be similarly assessed using the risk of bias 2 for cluster-randomized trials (RoB 2 CRT) (22). Additionally, we will use the Risk of Bias in Non-randomized Studies of Interventions (ROBINS-I) tool (23), to assess the risk of bias for controlled before-after studies and non-randomized controlled trials.

**Synthesis of evidence**

All included studies will be systematically synthesized in the text and a table following the SWiM guidelines (Synthesis Without Meta-analysis) (24). In this synthesis, we will describe how many sources of evidence were screened, assessed for eligibility, and included in the review, along with reasons for exclusion at each stage. Our presentation of included sources of evidence will include summary characteristics and citations, as well as a critical appraisal, if applicable. Studies will be grouped based on methods and interventions, standardized outcomes metrics, synthesis methods, criteria used to prioritize results for summary, reporting of results, the certainty of results, heterogeneity in effects, as well as barriers and facilitators to delivering the interventions will be discussed. For continuous outcomes, effect estimates will be expressed as mean differences (with 95% confidence intervals) between the intervention group and the control group; for dichotomous outcomes, effect estimates will be expressed as risk ratios, rate ratios, hazard ratios, or odds ratios (all with a 95% confidence interval). Additionally, we will discuss the



For peer review only - http://bmjopen.bmj.com/site/about/guidelines.xhtml

limitations of the review process and provide an interpretation of the results concerning the objectives of the review, as well as possible implications or next steps. We will follow the PRISMA Extension for Scoping Reviews (PRISMA-ScR) checklist and guidelines to ensure a robust and replicable process (20).

**Registration and reporting**

The final protocol was registered prospectively with the Open Science Framework (https://osf.io/t2d78) on July 19, 2023, based on the PRISMA Extension for Scoping Reviews (PRISMA-ScR) (20). In the event of protocol amendments, the date of each amendment will be accompanied by a description of each change and the rationale on the Open Science Forum.

**Ethics and dissemination**

This study is a scoping review that does not require ethics approval because it involves a methodical presentation of available resources. The protocol aims to provide an overview of the broad literature on community-based interventions targeting multiple forms of malnutrition among adolescents in LMICs. We anticipate that the findings of this review will be disseminated through publications in peer-reviewed journals, and presentations at international and regional conferences and stakeholder meetings targeting researchers, adolescents, policymakers, and governments in LMICs. Additionally, the submitted review will help identify effective interventions, determine gaps and disparities among interventions, and provide insight for policymakers to develop and design as well as implement future programs.

**Patient and public involvement**

None. This work analyses existing research studies, and therefore, involves no patients or members of the public.

**Author Contributors**

SS conceived the idea, developed the methods, and wrote the first draft of the manuscript. ALK, MR, UP, and EF contributed to the methods and supported the drafting and editing of the manuscript. ALK and MR



For peer review only - http://bmjopen.bmj.com/site/about/guidelines.xhtml

contributed meaningfully to the design of the search strategy. SO, NM, DOA, CN, SL, TB, WWF, and ARISE-NUTRINT study collaborators supervised and reviewed the protocol. All authors revised and approved the final manuscript.

**Funding**

This study was funded by the European Union Horizon 2022. Views and opinions expressed are however those of the author(s) only and do not necessarily reflect those of the European Union. Neither the European Union nor the granting authority can be held responsible for them.

**Competing interests**

None declared.

**Patient consent for publication**

Not required

**Provenance and peer review**

Not commissioned, externally peer-reviewed

**References**

1. United Nations Children's Fund. Progress for Children: A Report Card on Adolescents No. 10. 2012. Available at www.unicef.org/publications/files/Progress_for_Children_-_No._10_EN_04272012.pdf.
2. United Nations. Department of Economic and Social Affairs, Population Division. World population prospects, the 2012 revision. Available at: https://population.un.org/wpp/
3. Patton GC, Sawyer SM, Santelli JS, et al. Our future: a Lancet commission on adolescent health and well-being. Lancet 2016;387:2423-78.
4. Patton GC, Olsson C, Skirbekk V, et al. Adolescence and the next generation. Nature 2018;554: 458–66.

For peer review only

5. Canavan CR, Fawzi WW. Addressing knowledge gaps in adolescent nutrition: Towards advancing public health and sustainable development. Curr Dev Nutr 2019;3:nzz062.

6. World Health Organization. Global health estimates: leading causes of DALYs. Available at: https://www.who.int/data/gho/data/themes/mortality-and-global-health-estimates/global-health-estimates-leading-causes-of-dalys

7. Norris SA, Frongillo EA, Black MM, et al. Nutrition in adolescent growth and development. Lancet 2022;299:172-84.

8. Martin-Shields CP, Stojetz W. Food security and conflict: Empirical challenges and future opportunities for research and policy making on food security and conflict. World development 2019;119:150-64.

9. Salam RA, Hooda M, Das JK, et al. Interventions to Improve Adolescent Nutrition: A Systematic Review and Meta-Analysis. J Adolesc Health 2016;59:S29-S39.

10. Lassi ZS, Moin A, Das JK, et al. Systematic review on evidence-based adolescent nutrition interventions. Ann N Y Acad Sci 2017;1393:34-50.

11. Roche ML, Samson KLI, Green TJ, et al. Perspective: Weekly Iron and Folic Acid Supplementation (WIFAS): A critical review and rationale for inclusion in the essential medicines list to accelerate anemia and neural tube defects reduction. Adv Nutr 2021;12:334-42.

For peer review only



For peer review only - http://bmjopen.bmj.com/site/about/guidelines.xhtml

12. Shinde S, Wang D, Moulton GE, et al. School-based health and nutrition interventions addressing double burden of malnutrition and educational outcomes of adolescents in low- and middle-income countries: A systematic review. Matern Child Nutr 2023:e13437.

13. Kyere P, Veerman JL, Lee P, et al. Effectiveness of school-based nutrition interventions in sub-Saharan Africa: a systematic review. Public Health Nutr 2020;23:2626-36.

14. Van Cauwenberghe E, Maes L, Spittaels H, et al. Effectiveness of school-based interventions in Europe to promote healthy nutrition in children and adolescents: systematic review of published and 'grey' literature. Br J Nutr 2010;103:781-97.

15. Pongutta S, Ajetunmobi O, Davey C, et al. Impacts of school nutrition interventions on the nutritional status of school-aged children in Asia: A systematic review and meta-analysis. Nutrients 2022;14:589. doi: 10.3390/nu14030589.

16. Hawkes C, Ruel M, Salm L, et al. Double-duty actions: seizing programme and policy opportunities to address malnutrition in all its forms. Lancet 2019;395:142-55.

17. Munn Z, Peters MDJ, Stern C, et al. Systematic review or scoping review? Guidance for authors when choosing between a systematic or scoping review approach. BMC Med Res Methodol 2018 19;18:143.

18. Singh JA, Siddiqi M, Parameshwar P, et al. World Health Organization Guidance on Ethical Considerations in Planning and Reviewing Research Studies on Sexual and Reproductive Health in Adolescents. J Adolesc Health 2019;64:427-29.

19. World Bank designated developing countries 2023. World bank. Available at: https://www.endocrine.org/membership/join/developing-country-list



For peer review only - http://bmjopen.bmj.com/site/about/guidelines.xhtml

20. Tricco AC, Lillie E, Zarin W, et al. PRISMA Extension for Scoping Reviews (PRISMA-ScR): Checklist and Explanation. Ann Intern Med 2018;169:467-73.

21. Sterne JAC, Savović J, Page MJ, et al. RoB 2: a revised tool for assessing the risk of bias in randomized trials. BMJ 2019;366:l4898.

22. Eldridge S, Campbell M, Cambell M, et al. Revised Cochrane risk of bias tool for randomized trials (RoB 2.0) additional considerations for cluster randomized trials. Available at: https://www.unisa.edu.au/contentassets/72bf75606a2b4abcaf7f17404af374ad/rob2-0_cluster_parallel_guidance.pdf

23. Sterne JA, Hernán MA, Reeves BC, et al. ROBINS-I: a tool for assessing the risk of bias in non-randomized studies of interventions. BMJ 2016;355: i4919.

24. Campbell M, McKenzie JE, Sowden A, et al. Synthesis without meta-analysis (SWiM) in systematic reviews: reporting guideline. BMJ 2020;368:l6890.

For peer review only

**Supplementary File 1.** Search strategies in 3 different databases

**Community-based interventions targeting multiple forms of malnutrition among adolescents in low- and middle-income countries: protocol for a scoping review**

| No. | Concept | PubMed search terms | Number of records (As per 14th July) |
|---|---|---|---|
| #1 | **Randomized controlled trial Controlled before-after studies Quasi experimental studies** | ("randomized controlled trial"[pt] OR "random allocation"[mesh] OR "cross-over studies"[mesh] OR "Controlled Before-After Studies"[Mesh] OR quasi experiment*[tiab] OR quasiexperiment*[tiab] OR "quasi-experiment*"[tiab]) | 720,799<br>556,703 (2000-2023) |
| #2 | **Nutrition and health interventions** (dietary supplements, healthy diet, healthy eating, healthy nutrition, weight control, weight management, micronutrient supplementation diet/nutrition education, physical activity, community/home garden, and WASH, and nutrition policy) | ("Health Education"[Mesh:NoExp] OR "Health Promotion"[Mesh]) OR ("Adolescent Health Services"[Mesh] OR "Preventive Health Services"[Mesh:NoExp]) OR (preventive health[tiab]) OR ("Dietary Supplements"[Mesh] OR dietary supplement*[tiab] micronutrient supplement*[tiab] OR folic acid supplement*[tiab] OR MMN[tiab] OR nutrient supplement*[tiab] OR nutritional supplement*[tiab]) OR ("Diet, Healthy"[Mesh] OR healthy diet*[tiab] OR healthy eating[tiab] OR healthy food*[tiab] OR diet education[tiab] OR dietary education[tiab] OR dietary intervention[tiab] OR healthy eating[tiab] OR healthy food*[tiab] OR healthy diet*[tiab] OR healthy nutrition*[tiab] OR nutrition counsel*[tiab] OR nutritional counsel*[tiab]) OR (nutrition education[tiab] OR nutrition intervention[tiab] OR nutritional education[tiab] OR nutritional intervention[tiab]) OR ("exercise"[MeSH] OR "exercise" [tiab] OR "physical activity"[tiab] OR fitness*[tiab] OR sport*[tiab]) OR ("nutrition policy"[MeSH] OR "nutrition"[tiab] AND "policy"[tiab] OR "nutrition policy"[tiab]) | 865,050<br>687,256 (2000-2023) |
| #3 | **Adolescents** | ("Adolescent"[Mesh] OR adolescent[tiab] OR adolescents[tiab] OR adolescence[tiab] OR teen[tiab] OR teens[tiab] OR teenage*[tiab]) | 2,310,507<br>1,421,658 (2000-2023) |
| #4 | **Low- and middle-income countries** | (Afghanistan*[tiab] OR Albania*[tiab] OR Algeria*[tiab] OR Samoa*[tiab] OR Angola*[tiab] OR Armenia*[tiab] OR Azerbaijan*[tiab] OR Bangladesh*[tiab] OR Bengali[tiab] OR Belarus*[tiab] OR Belize[tiab] OR Benin[tiab] OR Bhutan*[tiab] OR Bolivia*[tiab] OR Bosnia*[tiab] OR Herzegovina*[tiab] OR Botswana*[tiab] OR Brazil*[tiab] OR Bulgaria*[tiab] OR "Burkina Faso"[tiab] OR Burkinabe[tiab] OR Burundi*[tiab] OR "Cabo Verd*"[tiab] OR "Cape | 1,931,426<br>1,565,369 (2000-2023) |

For peer review only

| | | | |
|---|---|---|---|
| | | Verd*"[tiab] OR Cambodia*[tiab] OR Cameroon*[tiab] OR "Central African*"[tiab] OR Chad*[tiab] OR China[tiab] OR Chinese[tiab] OR Colombia*[tiab] OR Comoros[tiab] OR Congo[tiab] OR "Costa Rica*"[tiab] OR "Cote d'Ivoire"[tiab] OR "Ivory Coast"[tiab] OR Cuba[tiab] OR Cuban[tiab] OR Djibouti[tiab] OR Dominica*[tiab] OR Ecuador[tiab] OR Egypt*[tiab] OR "El Salvador*"[tiab] OR Eritrea*[tiab] OR Ethiopia*[tiab] OR Fiji*[tiab] OR Gabon*[tiab] OR Gambia*[tiab] OR Georgia*[tiab] OR Ghana*[tiab] OR Grenada*[tiab] OR Guatemala*[tiab] OR Guinea*[tiab] OR Guyan*[tiab] OR Haiti*[tiab] OR Hondura*[tiab] OR India[tiab] OR Indian*[tiab] OR Indonesia*[tiab] OR Iran*[tiab] OR Iraq*[tiab] OR Jamaica*[tiab] OR Jordan*[tiab] OR Kazakh*[tiab] OR Kenya*[tiab] OR Kiribati[tiab] OR "People's Republic of Korea"[tiab] OR "North Korea"[tiab] OR Kosovo[tiab] OR Kosovar*[tiab] OR Kyrgyz*[tiab] OR Lao[tiab] OR Laos[tiab] OR Laotian*[tiab] OR Lebanon[tiab] OR Lebanes*[tiab] OR Lesotho[tiab] OR Liberia*[tiab] OR Libya*[tiab] OR Macedonia*[tiab] OR Madagascar*[tiab] OR Malawi*[tiab] OR Malaysia*[tiab] OR Maldives[tiab] OR Mali[tiab] OR "Marshall Island*"[tiab] OR "Mexico"[MeSH] OR Mexico[tiab] OR Mexican*[tiab] OR Micronesia*[tiab] OR Moldova*[tiab] OR Mongolia*[tiab] OR Montenegr*[tiab] OR Morocc*[tiab] OR Mozambique[tiab] OR Myanmar[tiab] OR Burmese*[tiab] OR Burma[tiab] OR Namibia*[tiab] OR Nepal*[tiab] OR Nicaragua*[tiab] OR Niger*[tiab] OR Pakistan*[tiab] OR Paraguay*[tiab] OR Peru*[tiab] OR Philippin*[tiab] OR Rwanda*[tiab] OR "Sao Tome"[tiab] OR Principe[tiab] OR Senegal*[tiab] OR Serbia*[tiab] OR "Sierra Leone*"[tiab] OR "Solomon Island*"[tiab] OR Somalia*[tiab] OR "South Africa*"[tiab] OR "Sri Lanka"[tiab] OR "St Lucia"[tiab] OR "Saint Lucia"[tiab] OR "St Vincent"[tiab] OR "Saint Vincent"[tiab] OR Grenad*[tiab] OR Sudan*[tiab] OR Suriname*[tiab] OR Swaziland*[tiab] OR Eswatini*[tiab] OR Syria*[tiab] OR Tajik*[tiab] OR Tanzania*[tiab] OR Zanzibar[tiab] OR Thai*[tiab] OR Timor*[tiab] OR Togo*[tiab] OR Tonga*[tiab] OR Tunisia*[tiab] OR Turkey[tiab] OR Turkish[tiab] OR Turkmen*[tiab] OR Tuvalu*[tiab] OR Uganda*[tiab] OR Ukrain*[tiab] OR Uzbeki*[tiab] OR Vanuatu*[tiab] OR Venezuela*[tiab] OR Vietnam*[tiab] OR "Viet nam*"[tiab] OR "West Bank"[tiab] OR Gaza*[tiab] OR Palestin*[tiab] OR Yemen*[tiab] OR Zambia*[tiab] OR Zimbabw*[tiab] OR "Western Sahara"[tiab] OR Argentin*[tiab] OR Russia*[tiab] OR Maurit*[tiab] OR Palau[tiab] OR Romania*[tiab]) | |
| | **#1 AND # 2 AND #3 AND #4** | | **10,670**<br>**9,869 (2000-2023)** |

For peer review only - http://bmjopen.bmj.com/site/about/guidelines.xhtml

| No. | Concept | EMBASE search terms | Number of records (As per 14th July) |
|---|---|---|---|
| #1 | **Randomized controlled trial Controlled before-after studies Quasi experimental studies** | ('randomized controlled trial':af OR 'randomization'/exp OR 'crossover procedure'/exp OR 'epidemiology'/exp OR quasi experiment*:ti,ab OR quasiexperiment*:ti,ab OR 'quasi-experiment*':ti,ab) | 146,384<br>131,436 (2000-2023) |
| #2 | **Nutrition and health interventions** (dietary supplements, healthy diet, healthy eating, healthy nutrition, weight control, weight management, micronutrient supplementation diet/nutrition education, physical activity, community/home garden, and WASH, and nutrition policy) | ('health education'/de OR 'health promotion'/exp) OR ('child health care'/exp OR 'preventive health service'/de) OR (preventive health:ti,ab) OR ('dietary supplement'/exp OR dietary supplement*:ti,ab micronutrient supplement*:ti,ab OR folic acid supplement*:ti,ab OR MMN:ti,ab OR nutrient supplement*:ti,ab OR nutritional supplement*:ti,ab) OR ('healthy diet'/exp OR healthy diet*:ti,ab OR healthy eating:ti,ab OR healthy food*:ti,ab OR diet education:ti,ab OR dietary education:ti,ab OR dietary intervention:ti,ab OR healthy eating:ti,ab OR healthy food*:ti,ab OR healthy diet*:ti,ab OR healthy nutrition*:ti,ab OR nutrition counsel*:ti,ab OR nutritional counsel*:ti,ab) OR (nutrition education:ti,ab OR nutrition intervention:ti,ab OR nutritional education:ti,ab OR nutritional intervention:ti,ab) OR ('exercise'/exp OR 'exercise':ti,ab OR 'physical activity':ti,ab OR fitness*:ti,ab OR sport*:ti,ab) OR ('nutrition policy'/exp OR 'nutrition':ti,ab AND 'policy':ti,ab OR 'nutrition policy':ti,ab) | 1,421,386<br>1,168,709 (2000-2023) |
| #3 | **Adolescents** | ('adolescent'/exp OR adolescent:ti,ab OR adolescents:ti,ab OR adolescence:ti,ab OR teen:ti,ab OR teens:ti,ab OR teenage*:ti,ab) | 2,031,554<br>1,398,667 (2000-2023) |
| #4 | **Low- and middle-income countries** | (Afghanistan*:ti,ab OR Albania*:ti,ab OR Algeria*:ti,ab OR Samoa*:ti,ab OR Angola*:ti,ab OR Armenia*:ti,ab OR Azerbaijan*:ti,ab OR Bangladesh*:ti,ab OR Bengali:ti,ab OR Belarus*:ti,ab OR Belize:ti,ab OR Benin:ti,ab OR Bhutan*:ti,ab OR Bolivia*:ti,ab OR Bosnia*:ti,ab OR Herzegovina*:ti,ab OR Botswana*:ti,ab OR Brazil*:ti,ab OR Bulgaria*:ti,ab OR 'Burkina Faso':ti,ab OR Burkinabe:ti,ab OR Burundi*:ti,ab OR 'Cabo Verd*':ti,ab OR 'Cape Verd*':ti,ab OR Cambodia*:ti,ab OR Cameroon*:ti,ab OR 'Central African*':ti,ab OR Chad*:ti,ab OR China:ti,ab OR Chinese:ti,ab OR Colombia*:ti,ab OR Comoros:ti,ab OR Congo:ti,ab OR 'Costa Rica*':ti,ab OR 'cote d`ivoire':ti,ab OR 'Ivory Coast':ti,ab OR Cuba:ti,ab OR Cuban:ti,ab OR Djibouti:ti,ab OR Dominica*:ti,ab OR Ecuador:ti,ab OR Egypt*:ti,ab OR 'El Salvador*':ti,ab OR Eritrea*:ti,ab OR Ethiopia*:ti,ab OR Fiji*:ti,ab OR Gabon*:ti,ab OR Gambia*:ti,ab OR Georgia*:ti,ab OR Ghana*:ti,ab OR Grenada*:ti,ab OR Guatemala*:ti,ab OR Guinea*:ti,ab OR | 2,466,552<br>2,047,567 (2000-2023) |

For peer review only

| | | | |
|---|---|---|---|
| | | Guyan*:ti,ab OR Haiti*:ti,ab OR Hondura*:ti,ab OR India:ti,ab OR Indian*:ti,ab OR Indonesia*:ti,ab OR Iran*:ti,ab OR Iraq*:ti,ab OR Jamaica*:ti,ab OR Jordan*:ti,ab OR Kazakh*:ti,ab OR Kenya*:ti,ab OR Kiribati:ti,ab OR 'People`s Republic of Korea':ti,ab OR 'North Korea':ti,ab OR Kosovo:ti,ab OR Kosovar*:ti,ab OR Kyrgyz*:ti,ab OR Lao:ti,ab OR Laos:ti,ab OR Laotian*:ti,ab OR Lebanon:ti,ab OR Lebanes*:ti,ab OR Lesotho:ti,ab OR Liberia*:ti,ab OR Libya*:ti,ab OR Macedonia*:ti,ab OR Madagascar*:ti,ab OR Malawi*:ti,ab OR Malaysia*:ti,ab OR Maldives:ti,ab OR Mali:ti,ab OR 'Marshall Island*':ti,ab OR 'Mexico'/exp OR Mexico:ti,ab OR Mexican*:ti,ab OR Micronesia*:ti,ab OR Moldova*:ti,ab OR Mongolia*:ti,ab OR Montenegr*:ti,ab OR Morocc*:ti,ab OR Mozambique:ti,ab OR Myanmar:ti,ab OR Burmese*:ti,ab OR Burma:ti,ab OR Namibia*:ti,ab OR Nepal*:ti,ab OR Nicaragua*:ti,ab OR Niger*:ti,ab OR Pakistan*:ti,ab OR Paraguay*:ti,ab OR Peru*:ti,ab OR Philippin*:ti,ab OR Rwanda*:ti,ab OR 'Sao Tome":ti,ab OR Principe:ti,ab OR Senegal*:ti,ab OR Serbia*:ti,ab OR 'Sierra Leone*':ti,ab OR 'Solomon Island*':ti,ab OR Somalia*:ti,ab OR 'South Africa*':ti,ab OR 'Sri Lanka':ti,ab OR 'St Lucia':ti,ab OR 'Saint Lucia':ti,ab OR 'St Vincent':ti,ab OR 'Saint Vincent':ti,ab OR Grenad*:ti,ab OR Sudan*:ti,ab OR Suriname*:ti,ab OR Swaziland*:ti,ab OR Eswatini*:ti,ab OR Syria*:ti,ab OR Tajik*:ti,ab OR Tanzania*:ti,ab OR Zanzibar:ti,ab OR Thai*:ti,ab OR Timor*:ti,ab OR Togo*:ti,ab OR Tonga*:ti,ab OR Tunisia*:ti,ab OR Turkey:ti,ab OR Turkish:ti,ab OR Turkmen*:ti,ab OR Tuvalu*:ti,ab OR Uganda*:ti,ab OR Ukrain*:ti,ab OR Uzbeki*:ti,ab OR Vanuatu*:ti,ab OR Venezuela*:ti,ab OR Vietnam*:ti,ab OR 'Viet nam*':ti,ab OR 'West Bank':ti,ab OR Gaza*:ti,ab OR Palestin*:ti,ab OR Yemen*:ti,ab OR Zambia*:ti,ab OR Zimbabw*:ti,ab OR 'Western Sahara':ti,ab OR Argentin*:ti,ab OR Russia*:ti,ab OR Maurit*:ti,ab OR Palau:ti,ab OR Romania*:ti,ab) | |
| | **#1 AND # 2 AND #3 AND #4** | | **17,874**<br>**16,942 (2000-2023)** |

| No. | Concept | Cochrane search terms | Number of records (As of July 14th 2023) |
|---|---|---|---|
| #1 | **Randomized controlled trial**<br>**Controlled before-after studies**<br>**Quasi experimental studies** | #1 "randomized controlled trial"<br>#2 [mh "random allocation"]<br>#3 {OR #1-#2}<br>#4 [mh "cross-over studies"]<br>#5 [mh "Controlled Before-After Studies"]<br>#6 {OR #4-#5}<br>#7 (quasi NEXT experiment*):ti,ab<br>#8 quasiexperiment*:ti,ab<br>#9 (quasi-experiment*):ti,ab<br>#10 {OR #7-#9} | 702,362<br>664,394<br>(2000-2023) |
| #2 | **Nutrition and health interventions**<br>(Dietary supplements, healthy diet, healthy eating, healthy nutrition, overweight, anaemia, obesity, weight control, weight management, micronutrient supplementation diet/nutrition education, school meal, physical activity, school garden, and WASH, and nutrition policy) | #11 [mh ^"health education"] OR [mh "health promotion"]<br>#12 [mh "Adolescent Health Services"] OR [mh ^"Preventive Health Services"]<br>#13 [mh "Adolescent Health Services"]<br>#14 [mh ^"Preventive Health Services"]<br>#15 [mh "Dietary Supplements"] OR dietary supplement*:ti,ab OR micronutrient supplement*:ti,ab OR folic acid supplement*:ti,ab OR folic acid supplement*:ti,ab OR MMN:ti,ab OR nutrient supplement*:ti,ab OR nutritional supplement*:ti,ab<br>#16 [mh "Diet, Healthy"] OR healthy diet*:ti,ab OR healthy eating:ti,ab OR healthy food*:ti,ab OR diet education:ti,ab OR dietary education:ti,ab OR dietary intervention:ti,ab OR healthy nutrition*:ti,ab OR nutrition counsel*:ti,ab OR nutritional counsel*:ti,ab<br>#17 nutrition education:ti,ab OR nutrition intervention:ti,ab OR nutritional education:ti,ab OR nutritional intervention:ti,ab<br>#18 [mh exercise] OR exercise:ti,ab OR physical activity:ti,ab OR fitness*:ti,ab OR sport*:ti,ab<br>#19 [mh "nutrition policy"] OR nutrition:ti,ab AND policy:ti,ab OR "nutrition policy":ti,ab<br>#20 {OR #11-#19} | 237.951<br>222,406<br>(2000-2023) |
| #3 | **Adolescents** | #21 [mh Adolescent] OR adolescent:ti,ab OR adolescents:ti,ab OR adolescence:ti,ab<br>#22 teen:ti,ab OR teens:ti,ab OR teenage*:ti,ab<br>#23 {OR #21-#22} | 146,504<br>115,839<br>(2000-2023) |
| #4 | **Low- and middle-income countries** | #24 (Afghanistan*):ti,ab OR (Albania*):ti,ab OR (Algeria*):ti,ab OR (Samoa*):ti,ab OR (Angola*):ti,ab<br>#25 (Armenia*):ti,ab OR (Azerbaijan*):ti,ab OR (Bangladesh*):ti,ab OR (Bengali):ti,ab OR (Belarus*):ti,ab<br>#26 (Belize):ti,ab OR (Benin):ti,ab OR (Bhutan*):ti,ab OR (Bolivia*):ti,ab OR (Bosnia*):ti,ab<br>#27 (Herzegovina*):ti,ab OR (Botswana*):ti,ab OR (Brazil*):ti,ab OR (Bulgaria*):ti,ab OR ("Burkina Faso"):ti,ab OR Burkinabe:ti,ab<br>#28 Burundi*:ti,ab OR (Cabo NEXT Verd*):ti,ab OR (Cape NEXT Verd*):ti,ab OR Cambodia*:ti,ab OR Cameroon*:ti,ab<br>#29 (Central African*):ti,ab OR (Chad*):ti,ab OR (China):ti,ab OR (Chinese):ti,ab OR (Colombia*):ti,ab | 128,522<br>124,301<br>(2000-2023) |

For peer review only

| | | | |
|---|---|---|---|
| | | #30 (Comoros):ti,ab OR (Congo):ti,ab OR ("Cook Islands"):ti,ab OR (Costa NEXT Rica*):ti,ab OR ("Cote d'Ivoire"):ti,ab<br>#31 ("Ivory Coast"):ti,ab OR (Cuba):ti,ab OR (Cuban):ti,ab OR (Djibouti):ti,ab OR (Dominica*):ti,ab<br>#32 (Ecuador):ti,ab OR (Egypt):ti,ab OR (El NEXT Salvador*):ti,ab OR (Eritrea*):ti,ab OR (Ethiopia*):ti,ab<br>#33 (Fiji*):ti,ab OR (Gabon*):ti,ab OR (Gambia*):ti,ab OR (Georgia*):ti,ab<br>#34 (Ghana*):ti,ab OR (Grenada*):ti,ab OR (Guadeloupe):ti,ab OR (Guatemala*):ti,ab<br>#35 (Guinea*):ti,ab OR (Guyan*):ti,ab OR (Haiti*):ti,ab OR (Hondura*):ti,ab OR (India):ti,ab<br>#36 (Indian*):ti,ab OR (Indonesia*):ti,ab OR (Iran*):ti,ab OR (Iraq*):ti,ab OR (Jamaica*):ti,ab<br>#37 (Jordan*):ti,ab OR (Kazakh*):ti,ab OR (Kenya*):ti,ab OR (Kiribati):ti,ab OR ("People's Republic of Korea"):ti,ab<br>#38 ("North Korea"):ti,ab OR (Kosovo):ti,ab OR (Kosovar*):ti,ab OR (Kyrgyz*):ti,ab OR (Lao):ti,ab<br>#39 (Laos):ti,ab OR (Laotian*):ti,ab OR (Lebanon):ti,ab OR (Lebanes*):ti,ab OR (Lesotho):ti,ab<br>#40 (Liberia*):ti,ab OR (Libya*):ti,ab OR (Macedonia*):ti,ab OR (Madagascar*):ti,ab OR (Malawi*):ti,ab<br>#41 (Malaysia*):ti,ab OR (Maldives):ti,ab OR (Mali):ti,ab OR (Marshall NEXT Island*):ti,ab<br>#42 [mh Mexico] OR Mexico:ti,ab OR (Mexican*):ti,ab<br>#43 (Micronesia*):ti,ab OR (Moldova*):ti,ab OR (Mongolia*):ti,ab OR (Montenegr*):ti,ab OR (Morocc*):ti,ab<br>#44 (Mozambique):ti,ab OR (Myanmar):ti,ab OR (Burmese*):ti,ab OR (Burma):ti,ab OR (Namibia*):ti,ab<br>#45 Nepal*:ti,ab OR Nicaragua*:ti,ab OR Niger*:ti,ab OR Pakistan*:ti,ab OR Paraguay*:ti,ab<br>#46 Peru*:ti,ab OR Philippin*:ti,ab OR Rwanda*:ti,ab OR "Sao Tome":ti,ab OR Principe:ti,ab OR Senegal*:ti,ab OR Serbia*:ti,ab<br>#47 (Sierra NEXT Leone*):ti,ab OR (Solomon NEXT Island*):ti,ab OR Somalia*:ti,ab OR (South NEXT Africa*):ti,ab OR "Sri Lanka":ti,ab<br>#48 "St Lucia":ti,ab OR "Saint Lucia":ti,ab OR "St Vincent":ti,ab OR "Saint Vincent":ti,ab OR Grenad*:ti,ab<br>#49 Sudan*:ti,ab OR Suriname*:ti,ab OR Swaziland*:ti,ab OR Eswatini*:ti,ab OR Syria*:ti,ab OR Tajik*:ti,ab OR Tanzania*:ti,ab<br>#50 Zanzibar:ti,ab OR Thai*:ti,ab OR Timor*:ti,ab OR Togo*:ti,ab OR Tonga*:ti,ab OR Tunisia*:ti,ab<br>#51 Turkey:ti,ab OR Turkish:ti,ab OR Turkmen*:ti,ab OR Tuvalu*:ti,ab OR Uganda*:ti,ab OR Ukrain*:ti,ab<br>#52 Uzbeki*:ti,ab OR Vanuatu*:ti,ab OR Venezuela*:ti,ab OR Vietnam*:ti,ab OR Viet nam*:ti,ab<br>#53 Palestin*:ti,ab OR Yemen*:ti,ab OR Zambia*:ti,ab OR Zimbabw*:ti,ab OR "Western Sahara":ti,ab<br>#54 Argentin*:ti,ab OR Russia*:ti,ab OR Maurit*:ti,ab OR Palau:ti,ab OR Romania*:ti,ab<br>#55 {OR #24-#54} | |
| | **#1 AND # 2 AND #3 AND #4** | (#3 OR #6 OR #10) AND #20 AND #23 AND #55 | **1,504**<br>**1,504**<br>**(2000-2023)** |

**Supplementary File 2.** Sample of extraction form

| | Publication details | | | | | | |
|---|---|---|---|---|---|---|---|
| Sr. No | Title | Journal/ source | Calendar year of publication | first author | corresponding author | contact author email | funding agency |
| | | | | | | | |
| | | | | | | | |

| Study Methods | | | | | | | | |
|---|---|---|---|---|---|---|---|---|
| Calendar year of study | Country | Study type | Study design | Target population (age, gender, socioeconomic status, etc.) | Sample size | Inclusion criteria | Exclusion criteria | Analysis methods |
| | | | | | | | | |
| | | | | | | | | |

| Intervention | | | | | | | | | | | |
|---|---|---|---|---|---|---|---|---|---|---|---|
| Intervention name | Timing of intervention | Duration of intervention | Guiding theory/ framework | Intervention topics | Intervention components/ activities | Frequency and duration of intervention activities | Intervention delivery mechanism | Intervention delivery agents | Selection, training, and supervision of delivery agents | Intervention coverage | Control group intervention |
| | | | | | | | | | | | |
| | | | | | | | | | | | |

| Outcome | | | | | | | |
|---|---|---|---|---|---|---|---|
| Outcome 1 | Timepoint for Outcome 1 assessment | Outcome 1 Methods of measurement | Outcome 1 measure (units and tools) | Outcome *X* | Timepoint for outcome X assessment | Outcome X methods of measurement | Outcome X measure (units and tools) |
| | | | | | | | |
| | | | | | | | |

For peer review only - http://bmjopen.bmj.com/site/about/guidelines.xhtml

| Results | | | |
|---|---|---|---|
| **Quantitative findings** | **Qualitative findings** | **Theory to explain the success** | **Theory to explain the failure** |
| | | | |
| | | | |

For peer review only

**PRISMA-P (Preferred Reporting Items for Systematic review and Meta-Analysis Protocols) 2015 checklist: recommended items to address in a systematic review protocol***

| Section and topic | Item No | Checklist item |
|---|---|---|
| **ADMINISTRATIVE INFORMATION** | | |
| Title: | | |
| Identification | 1a | Identify the report as a protocol of a systematic review<br>Title, p1: "Community-based interventions targeting multiple forms of malnutrition among adolescents in low- and middle-income countries: protocol for a scoping review" |
| Update | 1b | If the protocol is for an update of a previous systematic review, identify as such<br>NA |
| Registration | 2 | If registered, provide the name of the registry (such as PROSPERO) and registration number<br>Abstract, p4: "The final protocol was registered prospectively with the Open Science Framework on July 19, 2023 (https://osf.io/t2d78)." |
| Authors: | | |
| Contact | 3a | Provide name, institutional affiliation, e-mail address of all protocol authors; provide physical mailing address of corresponding author<br>Please see title pages, page 1-3 |
| Contributions | 3b | Describe contributions of protocol authors and identify the guarantor of the review<br>Page 15: "**Author Contributors**<br>SS conceived the idea, developed the methods, and wrote the first draft of the manuscript. ALK, MR, and UP contributed to the methods and supported the drafting and editing of the manuscript. ALK and MR contributed meaningfully to the design of the search strategy. SO, NM, DOA, CN, SL, TB, WWF, and ARISE-NUTRINT study collaborators supervised and reviewed the protocol. All authors revised and approved the final manuscript." |
| Amendments | 4 | If the protocol represents an amendment of a previously completed or published protocol, identify as such and list changes; otherwise, state plan for documenting important protocol amendments<br>NA |
| Support: | | |
| Sources | 5a | Indicate sources of financial or other support for the review<br>Page 16: "**Funding**<br>This study was funded by the European Union Horizon 2022. Views and opinions expressed are however those of the author(s) only and do not necessarily reflect those of the European Union. Neither the European Union nor the granting authority can be held responsible for them." |

For peer review only

| | | |
|---|---|---|
| Sponsor | 5b | Provide name for the review funder and/or sponsor<br>Page 16: "**Funding**<br>This study was funded by the European Union Horizon 2022. Views and opinions expressed are however those of the author(s) only and do not necessarily reflect those of the European Union. Neither the European Union nor the granting authority can be held responsible for them." |
| Role of sponsor or funder | 5c | Describe roles of funder(s), sponsor(s), and/or institution(s), if any, in developing the protocol<br>Page 16: "**Funding**<br>This study was funded by the European Union Horizon 2022. Views and opinions expressed are however those of the author(s) only and do not necessarily reflect those of the European Union. Neither the European Union nor the granting authority can be held responsible for them." |
| **INTRODUCTION** | | |
| Rationale | 6 | Describe the rationale for the review in the context of what is already known<br>Please see the Introduction, pages 6-8. |
| Objectives | 7 | Provide an explicit statement of the question(s) the review will address with reference to participants, interventions, comparators, and outcomes (PICO)<br>Page 8:<br>"The purpose of this scoping review is to comprehensively review the literature to describe community-based interventions that address the multiple forms of malnutrition such as obesity, overweight, underweight, wasting, stunting, anemia, and micronutrient deficiencies affecting adolescents in LMICs and describe the effects of these interventions on nutrition and health." |
| **METHODS** | | |
| Eligibility criteria | 8 | Specify the study characteristics (such as PICO, study design, setting, time frame) and report characteristics (such as years considered, language, publication status) to be used as criteria for eligibility for the review<br>Pages 9-12:<br>Please see Table 1 and section "Eligibility" |
| Information sources | 9 | Describe all intended information sources (such as electronic databases, contact with study authors, trial registers or other grey literature sources) with planned dates of coverage<br>Pages 8-9: "**Data sources, search terms and search strategy**<br>As part of our primary strategy, we will search MEDLINE (through PubMed), Embase, and CENTRAL (through the Cochrane Library). All databases will be searched for eligible studies from January 1, 2000, through July 14, 2023. We will identify potentially relevant published studies using the combination of medical subject headings (MeSH) and text words denoting nutrition-specific and nutrition-sensitive interventions. We will also examine references and bibliographies of included studies to identify additional sources of information. This search of studies will be supplemented by reviewing |

For peer review only

| | | |
|---|---|---|
| | | ClinicalTrials.gov and organizational websites such as the World Health Organization (WHO), World Bank, United Nations Children's Fund (UNICEF), and United Nations Population Fund (UNFPA). When possible, reports written in languages other than English will be translated by colleagues who are native speakers of those languages. No study will be considered if it cannot be adequately translated." |
| Search strategy | 10 | Present draft of search strategy to be used for at least one electronic database, including planned limits, such that it could be repeated<br>Please see Supplementary File 1 |
| Study records: | | |
| Data management | 11a | Describe the mechanism(s) that will be used to manage records and data throughout the review<br>Page 12: "**Data management**<br>The records will be imported into Covidence (Veritas Health Innovation, Melbourne, Australia), an Internet-based systematic review management program. Detection and removal of duplicates, title and abstract screening, and full-text screening will be performed by Covidence." |
| Selection process | 11b | State the process that will be used for selecting studies (such as two independent reviewers) through each phase of the review (that is, screening, eligibility and inclusion in meta-analysis)<br>Page 12: "**Selection of studies**<br>Using Covidence, we will screen titles, abstracts, and full texts. First, two reviewers will independently assess all search results (i.e., titles and abstracts) and exclude irrelevant studies based on inclusion and exclusion criteria. Next, two reviewers will carry out the full-text screening based on the same inclusion/exclusion criteria. The reviewers will discuss and resolve any difference of opinion or, if necessary, seek a third reviewer's opinion for resolving differences. A study flow diagram stating the specific reasons for exclusion will be maintained following the PRISMA for Scoping Review statement (PRISMA-ScR)." |
| Data collection process | 11c | Describe planned method of extracting data from reports (such as piloting forms, done independently, in duplicate), any processes for obtaining and confirming data from investigators<br>Pages 13: "**Data extraction**<br>Two reviewers will independently extract and enter data from studies included in the review. We will develop and test an extraction form on five randomly selected studies. We will extract the following information.<br>• Study details including the title, authors (first author and corresponding author), the corresponding author's contact information, journal (or source for unpublished reports), calendar year of publication, calendar year of intervention, country, and source of funding.<br>• Study methods including objectives and/or research questions, type of study, investigation strategies, settings, sample size, and sample characteristics (e.g., age, sex, socioeconomic status<br>• Intervention strategy including target population, delivery platform and providers (including selection, training, supervision, support, and incentivization), types of nutrition and other interventions (including content, conceptual framework and/or theoretical underpinnings, timing, duration, and dosage or frequency), |

For peer review only - http://bmjopen.bmj.com/site/about/guidelines.xhtml

| | | |
|---|---|---|
| | | and comparator/control.<br>• Outcomes assessed and details of the measures used.<br>• Findings including the coverage of services, facilitators and barriers to intervention delivery and uptake, effectiveness findings with point estimates and measures of variance (standard errors, 95% confidence intervals, or *p*-values), and any other key findings related to the scoping review questions.<br><br>We will contact the corresponding author via email if there is missing or inconsistent information. We will contact the author two times at most. The available data will be analyzed and any gaps due to missing data will be discussed if the data issue cannot be resolved after contacting the authors." |
| Data items | 12 | List and define all variables for which data will be sought (such as PICO items, funding sources), any pre-planned data assumptions and simplifications<br>Pages 13: "**Data extraction**<br>Two reviewers will independently extract and enter data from studies included in the review. We will develop and test an extraction form on five randomly selected studies. We will extract the following information.<br>• Study details including the title, authors (first author and corresponding author), the corresponding author's contact information, journal (or source for unpublished reports), calendar year of publication, calendar year of intervention, country, and source of funding.<br>• Study methods including objectives and/or research questions, type of study, investigation strategies, settings, sample size, and sample characteristics (e.g., age, sex, socioeconomic status<br>• Intervention strategy including target population, delivery platform and providers (including selection, training, supervision, support, and incentivization), types of nutrition and other interventions (including content, conceptual framework and/or theoretical underpinnings, timing, duration, and dosage or frequency), and comparator/control.<br>• Outcomes assessed and details of the measures used.<br>• Findings including the coverage of services, facilitators and barriers to intervention delivery and uptake, effectiveness findings with point estimates and measures of variance (standard errors, 95% confidence intervals, or *p*-values), and any other key findings related to the scoping review questions.<br><br>We will contact the corresponding author via email if there is missing or inconsistent information. We will contact the author two times at most. The available data will be analyzed and any gaps due to missing data will be discussed if the data issue cannot be resolved after contacting the authors." |
| Outcomes and prioritization | 13 | List and define all outcomes for which data will be sought, including prioritization of main and additional outcomes, with rationale<br>NA – Scoping Review |
| Risk of bias in individual studies | 14 | Describe anticipated methods for assessing risk of bias of individual studies, including whether this will be done at the |

| | | |
|---|---|---|
| | | outcome or study level, or both; state how this information will be used in data synthesis<br>Page 13-14: "**Risk of bias assessment**<br>As scoping reviews are exploratory in nature, risk of bias assessments are not typically required as part of the guidelines for scoping reviews [cite: https://bmcmedresmethodol.biomedcentral.com/articles/10.1186/s12874-018-0611-x]. However, we plan to assess the risk of bias among studies with an available quantitative measure as a preliminary way of contextualising the reported measures of impact on the outcomes reported. For the assessment of the risk of bias in the selected studies, we will use the Cochrane Collaboration's revised tool for assessing the risk of bias in randomized trials (RoB 2). Two reviewers will independently evaluate methodological quality. Any uncertainties or disagreements will be resolved by discussion or by a third reviewer, whenever needed. The tool is a domain-based evaluation, in which critical assessments for risk of bias are made separately for various domains, including the randomization process, deviation from intended interventions, missing outcome data, measurement of the outcome, and selective outcome reporting. The risk of bias in clustered trials will be similarly assessed using the risk of bias 2 for cluster-randomized trials (RoB 2 CRT). Additionally, we will use the Risk of Bias in Non-randomized Studies of Interventions (ROBINS-I) tool, to assess the risk of bias for controlled before-after studies and non-randomized controlled trials." |
| Data synthesis | 15a | Describe criteria under which study data will be quantitatively synthesised<br>NA – Scoping review |
| | 15b | If data are appropriate for quantitative synthesis, describe planned summary measures, methods of handling data and methods of combining data from studies, including any planned exploration of consistency (such as $I^2$, Kendall's $\tau$)<br>NA – Scoping review |
| | 15c | Describe any proposed additional analyses (such as sensitivity or subgroup analyses, meta-regression)<br>NA – Scoping review |
| | 15d | If quantitative synthesis is not appropriate, describe the type of summary planned<br>Pages 14-15: "**Synthesis of evidence**<br>All included studies will be systematically synthesized in the text and a table following the SWiM guidelines (Synthesis Without Meta-analysis). In this synthesis, we will describe how many sources of evidence were screened, assessed for eligibility, and included in the review, along with reasons for exclusion at each stage. Our presentation of included sources of evidence will include summary characteristics and citations, as well as a critical appraisal, if applicable. Studies will be grouped based on methods and interventions, standardized outcomes metrics, synthesis methods, criteria used to prioritize results for summary, reporting of results, the certainty of results, heterogeneity in effects, as well as barriers and facilitators to delivering the interventions will be discussed. For continuous outcomes, effect estimates will be expressed as mean differences (with 95% confidence intervals) between the intervention group and the control group; for dichotomous outcomes, effect estimates will be expressed as risk ratios, rate ratios, hazard ratios, or odds ratios (all with a 95% confidence interval). Additionally, we will discuss the limitations of the review process and provide an interpretation of the results concerning the objectives of the review, as well as possible implications or next steps. We will follow the PRISMA Extension for Scoping Reviews (PRISMA-ScR) checklist and guidelines to ensure a robust and replicable process." |

For peer review

| | | |
|---|---|---|
| Meta-bias(es) | 16 | Specify any planned assessment of meta-bias(es) (such as publication bias across studies, selective reporting within studies)<br>NA – Scoping Review |
| Confidence in cumulative evidence | 17 | Describe how the strength of the body of evidence will be assessed (such as GRADE)<br>NA – Scoping Review |

*** It is strongly recommended that this checklist be read in conjunction with the PRISMA-P Explanation and Elaboration (cite when available) for important clarification on the items. Amendments to a review protocol should be tracked and dated. The copyright for PRISMA-P (including checklist) is held by the PRISMA-P Group and is distributed under a Creative Commons Attribution Licence 4.0.**

*From: Shamseer L, Moher D, Clarke M, Ghersi D, Liberati A, Petticrew M, Shekelle P, Stewart L, PRISMA-P Group. Preferred reporting items for systematic review and meta-analysis protocols (PRISMA-P) 2015: elaboration and explanation. BMJ. 2015 Jan 2;349(jan02 1):g7647.*

For peer review only
